# Quantitative Structure–Mutation–Activity Relationship Tests (QSMART) model for protein kinase inhibitor response prediction

**DOI:** 10.1186/s12859-020-03842-6

**Published:** 2020-11-12

**Authors:** Liang-Chin Huang, Wayland Yeung, Ye Wang, Huimin Cheng, Aarya Venkat, Sheng Li, Ping Ma, Khaled Rasheed, Natarajan Kannan

**Affiliations:** 1 Institute of Bioinformatics, University of Georgia, 120 Green St., Athens, GA 30602 USA; 2 Department of Statistics, University of Georgia, 310 Herty Drive, Athens, GA 30602 USA; 3 Department of Biochemistry and Molecular Biology, 120 Green St., Athens, GA 30602 USA; 4 Department of Computer Science, 415 Boyd Graduate Studies Research Center, Athens, GA 30602 USA

**Keywords:** Protein kinase inhibitor, Precision medicine, Machine learning, Systems pharmacology

## Abstract

**Background:**

Protein kinases are a large family of druggable proteins that are genomically and proteomically altered in many human cancers. Kinase-targeted drugs are emerging as promising avenues for personalized medicine because of the differential response shown by altered kinases to drug treatment in patients and cell-based assays. However, an incomplete understanding of the relationships connecting genome, proteome and drug sensitivity profiles present a major bottleneck in targeting kinases for personalized medicine.

**Results:**

In this study, we propose a multi-component Quantitative Structure–Mutation–Activity Relationship Tests (QSMART) model and neural networks framework for providing explainable models of protein kinase inhibition and drug response ($\hbox {IC}_{50}$) profiles in cell lines. Using non-small cell lung cancer as a case study, we show that interaction terms that capture associations between drugs, pathways, and mutant kinases quantitatively contribute to the response of two EGFR inhibitors (afatinib and lapatinib). In particular, protein–protein interactions associated with the JNK apoptotic pathway, associations between lung development and axon extension, and interaction terms connecting drug substructures and the volume/charge of mutant residues at specific structural locations contribute significantly to the observed $\hbox {IC}_{50}$ values in cell-based assays.

**Conclusions:**

By integrating multi-omics data in the QSMART model, we not only predict drug responses in cancer cell lines with high accuracy but also identify features and explainable interaction terms contributing to the accuracy. Although we have tested our multi-component explainable framework on protein kinase inhibitors, it can be extended across the proteome to investigate the complex relationships connecting genotypes and drug sensitivity profiles.

## Background

Chemotherapy has served as standard care for cancer treatments for decades; however, the resistance of cancer cells to chemotherapy presents a major challenge in effectively treating cancer patients [1]. A major contributing factor in drug resistance [2], as well as drug sensitivity [3], is the accumulation of mutations in oncogenic proteins such as protein kinases, which are primary targets for cancer drugs [4]. Mutations in protein kinases can have varying impacts on drug sensitivity depending on the structural location of mutations. For example, non-small cell lung cancer (NSCLC) cells harboring the T790M mutations in the Epidermal Growth Factor Receptor (EGFR) are resistant to the cancer drug, gefitinib, whereas cells harboring the L858R mutation are hypersensitive to the same drug [5, 6]. In contrast, cells harboring the double mutant (T790M/L858R) are only resistant to gefitinib but not sensitive to it [7]. As mutations impact the efficacy of different cancer drugs, there is a need to incorporate structural knowledge in drug response prediction methods.

To identify molecular and genomic features associated with drug sensitivity and resistance in cancer cells, the Genomics of Drug Sensitivity in Cancer Project (GDSC) [8] recently screened the drug responses of 266 anticancer drugs against $\sim$ 1000 human cancer cell lines. Moreover, to broaden the pharmacologic annotation for human cancers, the Cancer Cell Line Encyclopedia (CCLE) [9] provided the pharmacological profiles of 24 drugs across 504 cancer cell lines. By utilizing these datasets, several prediction models were built to pursue a more accurate drug response estimation by different types of approaches, from traditional statistical models, network-based models, machine learning methods, to state-of-the-art neural networks (Table 1).

**Table 1:** Current drug response prediction approaches

Date	Author	Best model	Compared models	Cancer cell line features				Drug response		Validation	Performance
				EXP	MUT	CNV	Others	GDSC	CCLE		
2013/04/30	Menden et al. [10]	ANN	RF		$\checkmark$	$\checkmark$		$\checkmark$		8-fold CV	$\hbox {R}^{2} = 0.72$
2014/01/01	Jang et al. [11]	GLM	RF, SVM, PCA, PLS	$\checkmark$	$\checkmark$	$\checkmark$	CLS	$\checkmark$	$\checkmark$	5-fold CV	r = $\sim$ 0.5
2014/03/03	Geeleher et al. [12]	GLM		$\checkmark$				$\checkmark$		LOOCV	AUC = 0.81
2015/06/30	Dong et al. [13]	SVM		$\checkmark$					$\checkmark$	10-fold CV	Accuracy = $\sim$ 0.8
2015/09/29	Zhang et al. [14]	Network	EN	$\checkmark$				$\checkmark$	$\checkmark$	LOOCV	r = 0.6
2016/03/31	Gupta et al. [15]	SVM		$\checkmark$	$\checkmark$	$\checkmark$			$\checkmark$	LOOCV	r = 0.78
2016/09/01	Ammad-Ud-Din et al. [16]	Kernel	GLM				PWY	$\checkmark$		5-fold CV	$\rho$ = $\sim$ 0.22
2016/12/28	Nguyen et al. [17]	MANOVA	RF	$\checkmark$				$\checkmark$		10-fold CV	MCC = 0.18
2017/01/09	Stanfield et al. [18]	Network	Kernel		$\checkmark$		PPI	$\checkmark$	$\checkmark$	LOOCV	AUC = 0.881
2017/07/15	Ammad-Ud-Din et al. [19]	GLM	RF, SVM, PLS, SGL	$\checkmark$			PWY	$\checkmark$		LOOCV	$\rho$ = 0.375
2017/08/28	Geeleher et al. [20]	Ridge		$\checkmark$				$\checkmark$		10-fold CV	$\rho$ = 0.48
2017/09/12	Rahman et al. [21]	RF		$\checkmark$				$\checkmark$	$\checkmark$	3-fold CV	AUC = $\sim$ 0.3
2018/02/01	Ding et al. [22]	DNN	EN, SVM	$\checkmark$	$\checkmark$	$\checkmark$		$\checkmark$	$\checkmark$	25-fold CV	AUC = 0.87
2018/06/11	Chang et al. [23]	CNN	RF, SVM				SNP	$\checkmark$		5% leave-out	$\hbox {R}^{2}$ = 0.843
2018/07/01	Cichonska et al. [24]	Kernel		$\checkmark$		$\checkmark$	SNP, MET	$\checkmark$		10-fold CV	r = 0.858
2018/08/15	He et al. [25]	Kernel	RF, EN, Ridge	$\checkmark$				$\checkmark$		3-fold CV	Precision = $\sim$ 0.35
2018/09/14	Juan-Blanco et al. [26]	Network		$\checkmark$	$\checkmark$				$\checkmark$	LOOCV	AUC = $\sim$ 0.72
2018/09/14	Le and Pham [27]	Network	Kernel	$\checkmark$	$\checkmark$			$\checkmark$	$\checkmark$	5-fold CV	r = 0.804
2018/12/07	Liu et al. [28]	Network		$\checkmark$				$\checkmark$	$\checkmark$	10-fold CV	r = 0.73
2019/01/22	Wei et al. [29]	Network		$\checkmark$				$\checkmark$	$\checkmark$	LOOCV	r = 0.63
2019/01/31	Wang et al. [30]	EN		$\checkmark$			PWY		$\checkmark$	10-fold CV	MSE = $\sim$ 2.8
2019/01/31	Chiu et al. [31]	DNN	SVM, PCA, LR	$\checkmark$	$\checkmark$			$\checkmark$		10% leave-out	r = $\sim$ 0.86
2019/02/27	Li et al. [32]	Mixture	RF, GLM	$\checkmark$					$\checkmark$	20% leave-out	r = 0.882
2019/05/01	Yang et al. [33]	Network + SVM	Kernel		$\checkmark$	$\checkmark$	PPI, MET	$\checkmark$		5-fold CV	AUC = 0.788
2019/07/11	Lind and Anderson [34]	RF	ANN, SVM		$\checkmark$			$\checkmark$		5-fold CV	r = 0.86
2019/07/29	Liu et al. [35]	CNN	ANN		$\checkmark$	$\checkmark$		$\checkmark$		10% leave-out	$\hbox {R}^{2}$ = 0.826
2019/10/31	Manica et al. [36]	MCA + CNN	RF, SVM	$\checkmark$		$\checkmark$	PPI	$\checkmark$		5-fold CV	$\underline{\textit{R}^{\textit{2}}\,=\,0.86}$
2019/11/04	Oskooei et al. [37]	Network	RF, LR	$\checkmark$			PPI	$\checkmark$		30-fold CV	r = $\sim$ 0.9

The best performing method is highlighted in underlined

ANN, artificial neural network; AUC, area under the ROC curve; CCLE, Cancer Cell Line Encyclopedia; CLS, cancer classification; CNN, convolutional neural network; CNV, copy number variation; CV, cross-validation; EN, elastic net; EXP, gene expression; GDSC, Genomics of Drug Sensitivity in Cancer; GLM, generalized linear model, including ridge, elastic net, and lasso regression; DNN, deep neural networks; LOOCV, leave-one-out cross-validation; LR, linear regression; MCA, multiscale convolutional attentive; MCC, Matthews correlation coefficient; MET, methylation; MSE, mean squared error; MUT, gene-level mutation (i.e. whether the gene is mutated or not); PCA, principal component analysis; PLS, partial least squares; PPI, protein–protein interaction; PWY, pathway; r, Pearson correlation coefficient; $\hbox {R}^{2}$, coefficient of determination; RF, random forests; $\rho$, Spearman’s rank correlation coefficient; RNN, recurrent neural network; SGL, sparse group lasso; SNP, single nucleotide polymorphism; SVM, support vector machine

Despite progress in the development of computational methods for drug response prediction, existing methods do not have the sensitivity to achieve “precision” medicine goals. The prediction performances measured by the coefficient of determination ($\hbox {R}^{2}$) are in the range from 0.25 to 0.78. More recently, deep neural networks (DNN) with multiple hidden layers such as CDRscan [23], tCNNS [35], and MCA [36] have been proposed that achieve $\hbox {R}^{2}$ higher than 0.8 ($\hbox {R}^{2} = 0.84$, 0.83, and 0.86, respectively). However, most of the cancer cell line features used in previous studies are based on gene expression profiles and do not explicitly consider associations between drugs and the structural location of mutations (Table 1). Consequently, the molecular mechanisms of drug–protein interactions cannot be inferred from these models. The trade-off between prediction performance and explainability is also an issue for existing methods, such as CDRscan, tCNNS, and MCA, as they do not explicitly reveal the features that contribute to the observed prediction performance. Consequently, the Defense Advanced Research Projects Agency (DARPA) recently launched the Explainable Artificial Intelligence program (XAI) [38] to facilitate building explainable models while maintaining prediction performance.

**Fig. 1:**
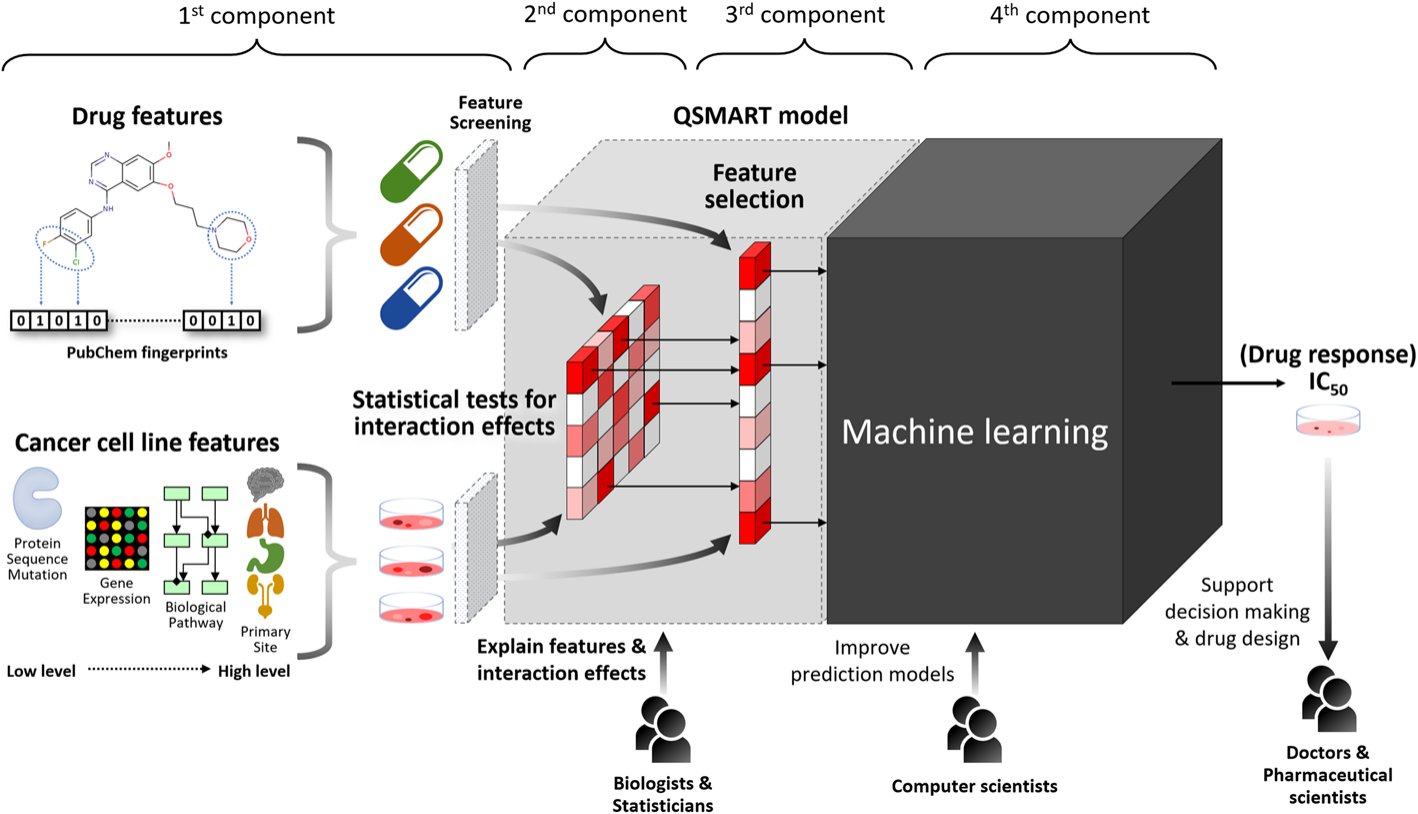
QSMART model with machine learning methods to predict protein kinase inhibitor response in cancer cell lines. Four main components of this framework: (1) drug and cancer cell line features, (2) statistical tests for interaction terms, (3) a feature selection method for identifying highly informative features, and (4) a machine learning method for predicting drug response

In recognition of the interest in building explainable AI models, we built the Quantitative Structure–Mutation–Activity Relationship Tests (QSMART) model, which extends the quantitative structure–activity relationship (QSAR) model to capture drug–mutation relationships. Additionally, it identifies the most informative drug and genomic features contributing to drug sensitivity predictions using traditional statistical and feature selection methods (Fig. 1). Although we cannot explain the entire model to humans in plain language, we show that these steps increase the prediction model’s explainability by moving two hidden layers outside the neural networks “black box”. The features and interaction terms in these two layers are interpretable by statisticians and biologists. When applied on a subset of protein kinase inhibitors (PKIs), the QSMART model achieves prediction accuracy comparable to or better than the state-of-the-art DNN methods (overall $\hbox {R}^{2} = 0.863$, AUC = 0.981, and RMSE = 0.811). Our studies represent the first systematic effort to develop explainable models for protein kinase inhibitor response prediction in cancer cell lines.

**Table 2:** Comparisons of drug response prediction by QSMART, DNN and statistical methods

Cancer type	${\#IC}_{{50}}$	QSMART model						Performance $({R}^{{2}})$					
		#All	#Drug	#Cancer features		#Interactions		QSMART + (NN/RF/SVM/EN)				Compared method	
		Features	Features	Residue	Others	DxM	Others	NN	RF	SVM	EN	$$\hbox {ANOVA}^{{*}}$$	$$\hbox {MCA}^{{**}}$$
AG	2971	62	31	0	9	4	18	0.879	0.588	0.581	0.293	0.672	0.656
Bone	3410	84	52	0	13	4	15	0.856	0.621	0.667	0.370	0.693	0.819
Breast	4706	129	70	5	26	12	16	0.880	0.604	0.673	0.496	0.702	0.814
CNS	4250	114	65	0	23	11	15	0.858	0.678	0.719	0.465	0.774	0.851
Cervix	1044	37	29	0	3	1	4	0.864	0.696	0.768	0.455	0.809	0.824
Endometrium	1073	33	21	0	4	4	4	0.878	0.596	0.580	0.328	0.769	0.832
Haematopoietic	4204	119	58	3	24	28	6	0.858	0.615	0.649	0.429	0.679	0.807
Kidney	2458	73	51	0	3	0	19	0.836	0.681	0.734	0.415	0.794	0.820
Large intestine	4628	141	53	10	14	50	14	0.814	0.617	0.692	0.495	0.736	0.794
Liver	1348	48	35	0	4	2	7	0.836	0.646	0.678	0.377	0.730	0.859
Lung (NSCLC)	9205	207	72	7	35	47	46	0.854	0.641	0.707	0.513	0.728	0.819
Lung (others)	7206	162	58	2	16	46	40	0.859	0.602	0.687	0.470	0.725	0.791
Lymphoid	13302	291	72	54	30	86	49	0.873	0.647	0.740	0.495	0.758	0.834
Oesophagus	3337	91	58	0	17	4	12	0.841	0.657	0.699	0.452	0.771	0.838
Ovary	3502	113	64	2	18	9	20	0.844	0.659	0.690	0.522	0.741	0.810
Pancreas	2421	84	60	0	7	0	17	0.833	0.693	0.737	0.492	0.784	0.816
Pleura	1431	36	23	0	5	0	8	0.805	0.629	0.623	0.303	0.776	0.837
Skin	5732	132	64	9	21	15	23	0.875	0.694	0.706	0.458	0.754	0.800
Soft tissue	1938	63	45	0	10	2	6	0.818	0.612	0.671	0.404	0.758	0.786
Stomach	2327	83	49	0	13	16	5	0.836	0.592	0.638	0.392	0.720	0.842
Thyroid	1352	33	25	0	5	0	3	0.830	0.644	0.680	0.398	0.798	0.853
UAT	3856	126	50	1	14	4	57	0.881	0.750	0.758	0.600	0.792	0.841
Urinary tract	1454	68	47	0	5	9	7	0.863	0.645	0.683	0.433	0.754	0.847
Overall	87155							0.863	0.655	0.710	0.460	0.755	0.823

The best performance for each cancer type is highlighted in underlined. The performance of each machine learning method is based on 10-fold cross-validation

$$\hbox {ANOVA}^{*}$$, analysis of variance, which did not undergo 10-fold cross-validation. $$\hbox {MCA}^{{**}}$$, multiscale convolutional attentive, a drug response prediction method [36]. The performance of MCA is based on its prediction for PKI response (Additional file 2). AG, autonomic ganglia; CNS, central nervous system; DxM, drug–mutation interaction term; EN, elastic net; NN, neural networks; NSCLC, non-small cell lung cancer; $$\hbox {R}^{2}$$, coefficient of determination; RF, random forests; SVM, support vector machine; UAT, upper aerodigestive tract; $$\#\hbox {IC}_{50}$$, the number of drug responses; #Nodes, the number of nodes in the first and second hidden layers of neural networks

## Results

### Performance of QSMART is comparable to DNN

**Fig. 2:**
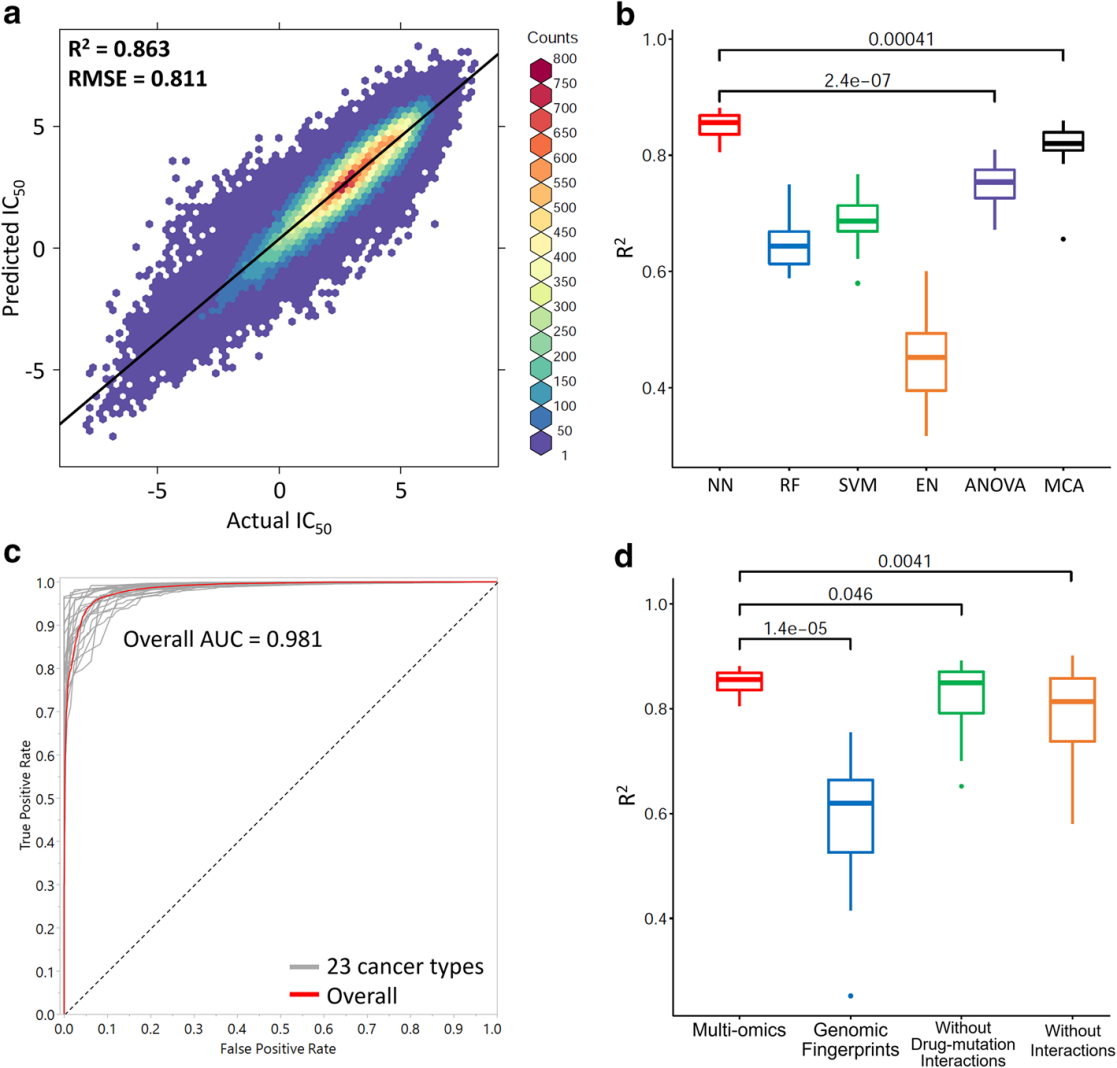
Prediction performances of different datasets and different prediction models. Wilcoxon signed-rank test is performed, and the *p* value is shown in each box plot. **a** Comparison between actual $\hbox {IC}_{50}$ (x-axis) and the $\hbox {IC}_{50}$ predicted by using QSMART with neural networks across all cancer types (y-axis). A fitted regression line is shown. **b** Prediction performances of different statistical or machine learning methods. NN: neural networks; RF: random forests; MCA: multiscale convolutional attentive [36]. **c** ROC curves for 23 cancer-centric models as well as an overall ROC. **d** Impact of different data sets on prediction performance

The QSMART model with neural networks predicts PKI responses in 23 cancer types with accuracies ranging from $\hbox {R}^{2} = 0.805$ to 0.881. Figure 2a presents $\hbox {IC}_{50}$ versus predicted $\hbox {IC}_{50}$ plot for all types of cancer cell lines (overall $\hbox {R}^{2} = 0.863$ and RMSE = 0.811). For each cancer-centric model, Table 2 summarizes the number of PKI responses, the total number of features (including drug features, cancer cell line features, and interaction terms), the number of nodes in the first and second hidden layers of neural networks, and prediction performance ($\hbox {R}^{2}$). Additional file 1: Table S1 shows additional measurements of prediction performance (RMSE and AUC), cancer cell line features at seven feature levels, interaction terms, and training iterations. Compared with commonly used machine learning models and a state-of-the-art DNN model, multiscale convolutional attentive (MCA) [36], the QSMART model with neural networks shows higher or comparable performances of predicting PKI response for 23 cancer types based on 10-fold cross-validation (Fig. 2b and Table 2). In this study, we designed three types of neural network architectures: single-layer, double-layer, and complex-double-layer. However, we found that the prediction models for all the 23 cancer types can achieve $\hbox {R}^{2}$ > 0.8 by using either single-layer or double-layer architecture. As per Occam’s razor principle, we only used the single-layer or double-layer architecture since they are able to achieve accuracies comparable to or better than the state-of-the-art DNN methods.

To further confirm the QSMART model’s ability to classify drug responses into two categories (sensitive versus non-sensitive), we chose thresholds to define actual $\hbox {IC}_{50}$ as sensitive or non-sensitive. Compared to a single threshold used in a previous study [23] ($\hbox {IC}_{50} = -\,2$), we set multiple thresholds ($-\,4, -\,3, -\,2, -\,1$, and 0) and averaged the results to avoid overestimating the prediction performance. The resulting ROC curves for 23 cancer types and the overall curve are shown in Fig. 2c. The overall AUC is 0.981 and comparable to a recent DNN-based study [23] (AUC > 0.98). AUC for each cancer type is available in the Additional file 1: Table S1.

### Multi-omics data are informative in prediction models

To investigate the extent to which multi-omics features introduced in this study contribute to drug response prediction, we compared the contribution of multi-omics features with simple genomic features such as genomic fingerprints. Genomic fingerprints are binary vectors representing genomic mutation positions. They are the only cancer cell line features used in one of the top-performing methods [23]. Thus we replaced our multi-omics cancer cell line features with 44,364 genomic fingerprints (Additional file 1: Figure S1) and ran our predictions with the same number of features, feature selection methods, and neural network architectures. The number of selected features, including interaction terms, and prediction performances are shown in Additional file 1: Table S2. The box plot in Fig. 2d shows that the performance distribution of 23 cancer-centric models using multi-omics features is significantly higher than that of the models using genomic fingerprints alone (overall $\hbox {R}^{2}$ = 0.863 versus 0.655, *p* value = 1.4e−05, Wilcoxon signed-rank test).

### Contribution of interaction terms in prediction models

We next wanted to evaluate the contribution of interaction terms (the second component in Fig. 1) in drug response prediction. We examined the prediction performance by removing drug–mutation interaction terms and removing all interaction terms. We utilized the feature selection method to prioritize all input features, selected the same total number of features in the original models shown in Table 2, and then used the same neural network architectures to train the new models. The results of these two experiments are shown in Additional file 1: Table S3 and Table S4, respectively. The box plot in Fig. 2d shows that the performance of the full QSMART model is better than the models without drug–mutation interaction terms (overall $\hbox {R}^{2}$ = 0.863 versus 0.846, *p* value = 0.046) and the models without any interaction terms (overall $\hbox {R}^{2}$ = 0.863 versus 0.817, *p* value = 0.0041). Intriguingly, for some cancer types, such as breast, models without any interaction terms achieve better performance than the QSMART model. This is likely because some more informative high-order interactions (three-way or even multi-way interactions), which cannot be detected by the statistical method we used, were captured inside the neural network black box and thus compensated for the lack of interaction terms in the input layer. However, neural networks cannot guarantee that these informative but unexplainable high-order interactions will always be captured under the limited number of samples and the training iteration we used. This fact is reflected in Fig. 2d, which shows that the prediction performance is variable when the drug–mutation interaction terms are eliminated ($\hbox {R}^{2}$ = 0.653 to 0.892), or all interaction terms are eliminated ($\hbox {R}^{2}$ = 0.581 to 0.901).

### Case study: non-small cell lung cancer

We next evaluated the contribution of different features in drug response prediction using non-small cell lung cancer (NSCLC) as a case study. All 207 features in the NSCLC-specific QSMART model and their descriptions are listed in Additional file 3. We choose several pertinent features and explain their biological relevance in this case study to demonstrate how scientists may use our prediction model to explain their findings.

#### Batch effects are significant factors influencing drug response

We first wanted to evaluate how drug response datasets generated from different sources contribute to drug response prediction. To this end, we introduced a feature termed “From_Sanger” in the model to distinguish the assays done by the Wellcome Sanger Institute (1) from the Massachusetts General Hospital (0). On average, the PKI responses obtained from Massachusetts General Hospital showed lower drug sensitivity (higher $\hbox {IC}_{50}$ value) than those from the Wellcome Sanger Institute in the NSCLC dataset (average actual $\hbox {IC}_{50}$ = 2.88 versus 2.41, *p* value = 1.3e−23, Wilcoxon rank-sum test). To investigate these experimental batch effects, we increased the value of “From_Sanger” by one unit and held other features constant. If we replace 0 with 1 for the “From_Sanger” feature, the average $\hbox {IC}_{50}$ predicted by the pre-trained model reduces to 0.65 (average predicted $\hbox {IC}_{50}$ = 2.87 versus 2.22, Additional file 3). Notably, this feature is selected not only in the NSCLC model but also in the other 22 cancer-centric models, implying that batch effects are significant factors for drug response prediction.

#### Contribution of Gene Ontology terms in drug response prediction

Next, we wanted to investigate how biological process interactions can contribute to drug response prediction. A biological process interaction term “GO_0030324_X_GO_0048675” is selected in the NSCLC model. This feature represents the product of the number of mutations perturbing the biological process “lung development” (Gene Ontology ID: GO:0030324) and the number of mutations perturbing “axon extension” (Gene Ontology ID: GO:0048675). Axon initiation, extension, and guidance are known to play essential roles in cancer invasion and metastasis [39]. In the NSCLC dataset, there are eight cell lines with mutations in protein kinases associated with axon extension; among them, NCI-H1944 and NCI-H2030 are from patients with metastatic NSCLC. On average, the NSCLC cell lines with “GO_0030324_X_GO_0048675” interaction showed higher PKI responses than those without this interaction (average actual $\hbox {IC}_{50}$ = 4.32 versus 2.69, *p* value = 1.4e−27, Wilcoxon rank-sum test). Comparatively, the NSCLC cell lines with mutations involved in “lung development” or “axon extension” alone showed lower PKI responses (average actual $\hbox {IC}_{50}$ = 3.20 or 2.07, respectively). Based on our prediction model, every unit increase in the interaction term “GO_0030324_X_GO_0048675” is associated with a 0.45 unit increase in $\hbox {IC}_{50}$ on average (average predicted $\hbox {IC}_{50}$ = 2.73 versus 3.18). This suggests that the lower PKI sensitivity for the NSCLC cell lines is likely due to mutations in genes involved in lung development (e.g., PDGFRA) and axon extension pathway (e.g., DCLK1 or ULK2).

#### Example of how PPIs contribute to drug response

**Fig. 3:**
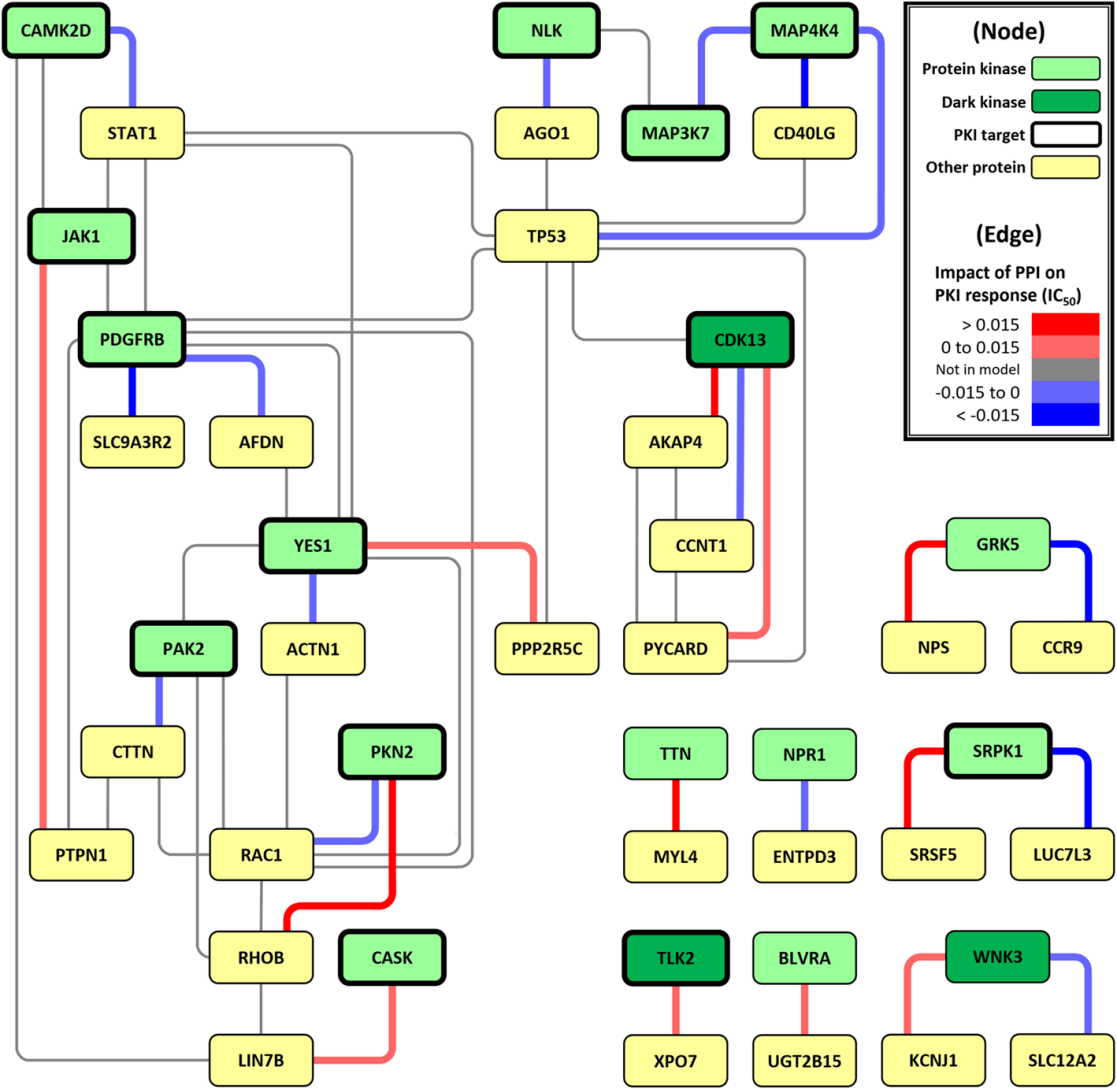
PPI network constructed by the interaction terms for predicting PKI response in NSCLC cell lines. Green node: protein kinase; dark green node: dark/understudied protein kinase [77]; yellow node: other protein; the node with a thick border: known PKI target; red edge: PPI with a positive impact on $\hbox {IC}_{50}$; light red edge: PPI with a weak positive impact on $\hbox {IC}_{50}$; blue edge: PPI with a negative impact on $\hbox {IC}_{50}$; light blue edge: PPI with a weak negative impact on $\hbox {IC}_{50}$; gray edge: PPI not in the prediction model

The NSCLC model contains 27 protein–protein interaction (PPI) terms. We quantify each PPI by the product of the gene expression level of individual proteins in the complex. Every unit of gene expression level increase in these 27 PPIs contributes to -0.089 to 0.061 unit increase in $\hbox {IC}_{50}$ on average. Gene enrichment analysis of the 27 genes in the TP53-centric subnetwork (shown in Fig. 3) revealed an overrepresentation of pathways associated with angiogenesis, inflammation, apoptosis, and axon guidance (Additional file 1: Table S5, performed by PANTHER [40]). MAP4K4 is one of the genes involved in the apoptosis signaling pathway, and its over-expression is a prognostic factor for lung adenocarcinoma [41]. MAP4K4 expression is up-regulated upon binding to p53, resulting in the activation of the apoptotic JNK signaling pathway [42]. In the NSCLC dataset, when the expression of MAP4K4-TP53 interaction (“EXP_MAP4K4_X_EXP_TP53”) increases, the average $\hbox {IC}_{50}$ is slightly decreased (Pearson correlation = − 0.10). In the pre-trained PKI response prediction model, every unit of gene expression level increase in MAP4K4-TP53 PPI is associated with a 0.012 unit decrease in $\hbox {IC}_{50}$ on average (average predicted $\hbox {IC}_{50}$ = 2.727 versus 2.715), suggesting that this up-regulated PPI in apoptotic JNK signaling pathway contributes causatively to the observed drug sensitivity.

#### Role of drug–mutation association in drug response prediction

**Fig. 4:**
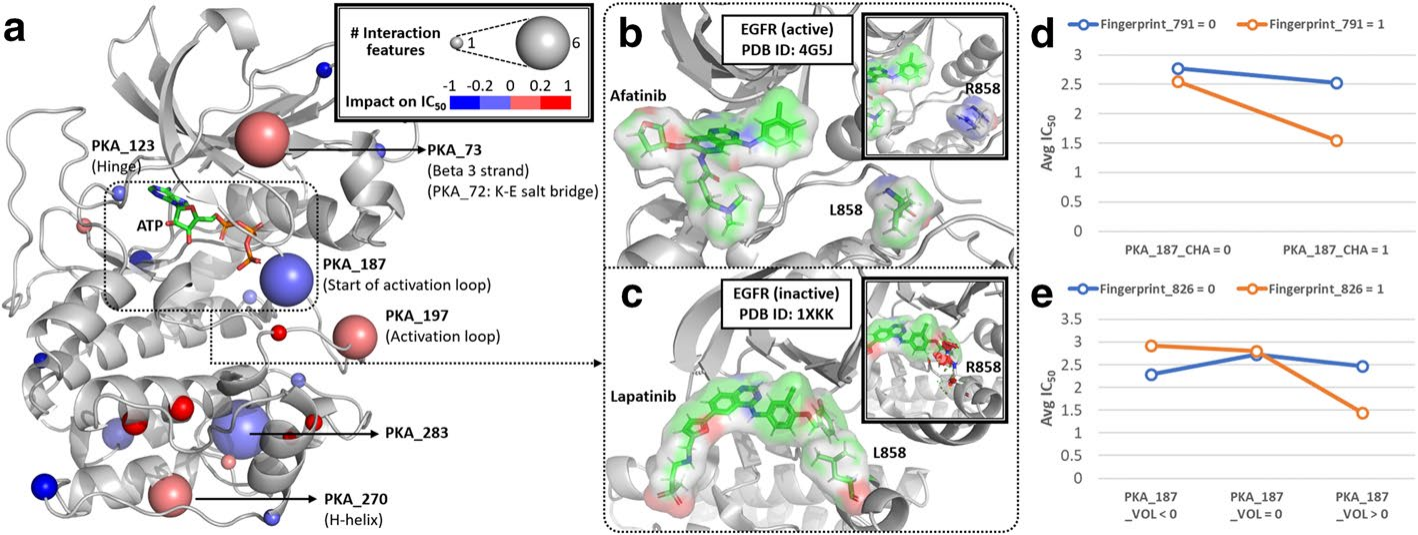
Distribution of drug–mutation relationships on the reference protein kinase A (PKA) crystal structure and interaction analyses. **a** Interaction hot spots are labeled and represented by larger spheres on the reference PKA structure (PDB ID: 1ATP). If a residue is involved in multiple drug–mutation relationships, the median of their impacts on $\hbox {IC}_{50}$ is chosen to represent the color of the sphere. Red sphere represents drug–mutation relationship with a positive impact on $\hbox {IC}_{50}$; blue sphere represents relationship with a negative impact on $\hbox {IC}_{50}$. **b**, **c** represent examples of two PKIs (afatinib and lapatinib) with different binding modes in the active (PDB ID: 4G5J) and inactive (PDB ID: 1XKK) conformations of EGFR, respectively. The residue corresponding to EGFR L858 (PKA_187) is labeled in each example; the mutant form (arginine) modeled in PyMol [78] is shown. **d**, **e** represent statistical interaction analyses for Fingerprint_791 versus PKA_187_CHA and Fingerprint_826 versus PKA_187_VOL in the NSCLC dataset, respectively

Finally, we wanted to investigate the extent to which drug–mutation interactions quantitatively contribute to PKI response prediction in NSCLC. In total, there are 47 drug–mutation interaction terms in the NSCLC model, and they are located at 22 structural locations represented by spheres in Fig. 4a (PDB ID: 1ATP). Their impacts on $\hbox {IC}_{50}$ are listed in Additional file 1: Table S6, sorted by absolute $\hbox {IC}_{50}$ impact. The drug–mutation relationships located in the canonical ATP-binding pocket (highlighted by a dashed rectangle in Fig. 4a) could be formed by type I or type II protein kinase inhibitors that bind to active or inactive kinase conformations, respectively [43]. For example, mutation mapping to the beginning of the activation segment (residue position 187 in protein kinase A (“PKA_187”) is located in this pocket. In the NSCLC dataset, there are three mutations located in PKA_187: EGFR L858R, BRAF L597V, and STK32C I237V.

Figure 4b, c respectively show different binding modes of two EGFR inhibitors (afatinib and lapatinib) that contribute to variable response in L858R mutant EGFR. H3255, an NSCLC cell line with EGFR L858R mutation, is hypersensitive to afatinib ($\hbox {IC}_{50}\,=\,-\,4.35$; average $\hbox {IC}_{50}$ = 2.03 for all the NSCLC cell lines treated with afatinib). Notably, the L858R mutation can be accommodated in the active conformation of EGFR, but not in the inactive state due to steric hindrance [44].

An interaction analysis (Fig. 4d) shows that the mutated residues involving charge difference at PKA_187 have significant interaction (*p* value = 0.043, F-test) with Fingerprint_791, a drug substructure “NC1CCC(N)CC1” of afatinib. Based on our prediction model, every unit increase in “PKA_187_CHA_X_Fingerprint_791”, an interaction term with one of the highest impact on $\hbox {IC}_{50}$ among all the drug–mutation interaction terms in the model (Additional file 1: Table S6), is associated with a 0.46 unit decrease in $\hbox {IC}_{50}$ on average (average predicted $\hbox {IC}_{50}$ = 2.73 versus 2.27). Another interaction analysis (Fig. 4e) shows that the mutated residues involving volume difference at PKA_187 have significant interaction (*p* value = 0.035, F-test) with Fingerprint_826, a drug substructure “OC1C(N)CCCC1” of afatinib. Every unit increase in “PKA_187_VOL_X_Fingerprint_826” is associated with a 0.01 unit decrease in $\hbox {IC}_{50}$ on average (average predicted $\hbox {IC}_{50}$ = 2.73 versus 2.72). Since lapatinib lacks both substructures Fingerprint_791 and Fingerprint_826, we speculate that mutant EGFR in NSCLC cells with a larger, positively charged mutation at PKA_187 are resistant to lapatinib (the blue lines in Fig. 4d, e).

## Discussion

In this study, we propose a PKI response prediction framework to estimate $\hbox {IC}_{50}$ values with a more explainable AI model. This framework includes four components: (1) drug features and cancer cell line’s multi-omics features, (2) statistical tests for capturing interaction effects, (3) feature selection, and (4) machine learning methods. We validated the contribution of each component and used the NSCLC dataset as a case study to explain the contributing features in PKI response prediction.

The intrinsic limitation of drug response prediction is the unexplainable variation of drug response caused by different assays and experimental conditions. Several previous studies on drug response prediction used data not only from GDSC but also from CCLE (Table 1). However, Juan-Blanco et al. [26] pointed out that although GDSC and CCLE datasets shared 343 cancer cell lines and 15 drugs, the drug responses from these two datasets were poorly correlated. Thus, we only used a single source in this study to minimize the unexplainable effect from different experimental conditions. Nevertheless, this situation impeded us from finding appropriate independent testing set outside the GDSC data. Even though the drug response data we used were only from GDSC, the feature selection process showed that the drug feature “From_Sanger” was selected for all the 23 cancer-centric prediction models, meaning that the batch effects are significant depending on the origin of datasets (Wellcome Sanger Institute vs. Massachusetts General Hospital). The GDSC 8.0 dataset was released while our studies were underway. Compared with release 7.0, it contains 160 thousand more drug responses. However, this dramatic increase does not provide us with an appropriate test set, because the old drug response dataset (called GDSC1 in release 8.0) and the new drug response dataset (called GDSC2) were generated based on different experimental protocols. Furthermore, PKI responses in the two datasets show a weak correlation ($\hbox {R}^{2}$ = 0.6, Additional file 1: Figure S2).

Our study has revealed different interaction terms and types contributing to the prediction of drug response profiles in cell-based assays. The QSMART model can potentially be extended to other applications, such as protein–ligand interaction, gene–environment interaction, and agent–host interaction. However, in addition to the unexplainable variation issue mentioned above, improving generalization performance is challenging for prediction models with multiple interaction terms, which require more samples to detect significant interactions [45]. We randomly removed 10% of the samples and compared the selected features of these reduced training sets with those of full training sets. We found that the full training sets’ 1896 (81.4%) features, including 75.4% of the interaction terms, were still selected in the reduced sets (Additional file 1: Table S7). The features discussed in the Case study, “From_Sanger”, “GO_0030324_X_GO_0048675”, “EXP_MAP4K4_X_EXP_TP53”, and “PKA_187_VOL_X_Fingerprint_826” were still selected in the reduced NSCLC set. Although “PKA_187_CHA_X_Fingerprint_791” was not selected, a relevant interaction term about the polarity change “PKA_187_POL_X_Fingerprint_791” was in the reduced NSCLC set. Nevertheless, 164 interaction terms were uniquely selected in the reduced sets. These unique interaction terms also showed statistical significance to drug response prediction in the full sets, but the feature selection methods did not select them under the BIC control. Although the number of training samples was reduced, more than three-quarters of the features were still in the models, and the overall performance did not significantly change (Additional file 1: Table S8). To increase generalization performance and the stability of our prediction framework, increasing the sample size will help. Thus, when people apply the concept of QSMART to other interaction types, sample size and sample availability should be considered.

## Conclusions

In conclusion, by integrating multi-omics data in the QSMART model, we not only predict PKI responses in cancer cell lines with high accuracy but also identify features and interaction terms contributing to the accuracy, thereby enhancing the explainability of the prediction models. Compared to traditional QSAR models, the QSMART model proposed in this study further introduces different types of interaction terms, which are usually hidden in deep neural network models. While we demonstrate our model in protein kinase inhibitor binding, the QSMART model can be applied to other druggable gene families such as G protein-coupled receptors (GPCRs).

## Methods

### Framework for drug response prediction

The overall objective of this study is to emphasize the contribution of interaction terms that capture drug–mutation relationships and to show how these interaction terms could help explain the mechanism of drug response. The framework we propose in this study includes four main components: (1) the substructure fingerprints of protein kinase inhibitor (PKI) and cancer cell line’s multi-omics features, including from low-level features, such as residue mutations, to high-level features, such as perturbed biological processes, (2) F-test for identifying significant drug–mutation relationships and other interaction effects, (3) a feature selection method: Lasso with Bayesian information criterion (BIC) control, and (4) a machine learning method to predict PKI response: neural networks (Fig. 1). The modular nature of this framework provides flexibility by allowing each component to be updated independently based on new datasets and methodology. To implement this framework, we collected a dataset containing  0.2 million drug responses ($\hbox {IC}_{50}$ in a logarithmic scale; “$\hbox {IC}_{50}$” hereinafter) from GDSC, split them into 23 sub-datasets according to the primary site where the cancer cell line originated, and then built a cancer-centric model for each sub-dataset. More details about each component are described below.

### Protein kinase inhibitor

We define small-molecule (molecular weight < 900 daltons) protein kinase inhibitors in GDSC from a variety of publicly available, manually curated drug-target databases, and experimental data. The list of human protein kinases in this study is defined by ProKinO [46] (version 2.0). Drug-kinase associations were extracted from DrugBank [47] (version 5.1.0), Therapeutic Target Database (TTD [48], last accessed on September 15th, 2017), Pharos [49] (last accessed on May 15th, 2018), and LINCS Data Portal [50] (last accessed on May 15th, 2018). We define a drug as a PKI if it is annotated as an “inhibitor”, “antagonist”, or “suppressor” in the drug–kinase associations. We also include the PKIs in LINCS Data Portal if their controls are less than 5% in $\hbox {KINOMEscan}^{\textregistered }$ assays. Based on these criteria, we define 143 small-molecule PKIs out of the 252 unique screened compounds in GDSC (Additional file 4).

### Drug response

GDSC (release 7.0) provides the half-maximal inhibitory concentration values ($\hbox {IC}_{50}$) for 224,202 drug-cancer cell line pairs. The drug sensitivity assays were performed either by the Wellcome Trust Sanger Institute or the Massachusetts General Hospital Cancer Center. In this drug response dataset, there are 12,509 duplicate drug-cancer cell line pairs due to 16 duplicate drugs. We measured the Pearson correlation coefficient between the $\hbox {IC}_{50}$ values of each duplicate drug in the two assays. Only afatinib and refametinib showed a strong positive correlation (r > 0.7); their $\hbox {IC}_{50}$ values were then merged by their weighted means [51]. We exclude duplicate drugs with a correlation coefficient of less than 0.7 from our study. The resulting dataset of 197,459 non-redundant drug responses consists of 236 drugs and 1065 cancer cell lines. After filtering out non-PKIs, 109,856 non-redundant drug responses consisting of 135 PKIs and 1064 cancer cell lines remained.

### Drug features

The 2D structures of drugs were obtained from PubChem in SDF format. The Chemistry Development Kit Descriptor Calculator Graphical User Interface [52] (version 1.4.6) generated 881 PubChem fingerprints and 286 chemical descriptors, including constitutional, topological, electronic, geometric, and bridge descriptors. Observing high redundancy and multicollinearity within features, we removed redundant features and implemented the variance inflation factor criterion (VIF) [53] to reduce multicollinearity (for more details, see the Feature screening section). After filtering, 92 PubChem fingerprints and 0 chemical descriptors remained.

### Cancer cell line features

Using mutation profiles for each cancer cell line sample provided by COSMIC Cell Lines Project [54] (v87), we incorporate 7 categories of multi-omics features to quantify the differences between wild type and mutant protein kinases:

1. Residue-level: reference protein kinase A (PKA) position (from ProKinO), mutant type, charge, polarity, hydrophobicity, accessible surface area, side-chain volume, energy per residue [55], and substitution score (BLOSUM62 [56])
2. Motif-level: sequence and structural motifs of protein kinase (from ProKinO)
3. Domain-level: subdomain in protein kinase (from ProKinO) and functional domain (from Pfam [57] v31.0)
4. Gene-level: the number of mutations in the genes encoding protein kinases, gene expression (from GDSC), and copy number variation (from COSMIC)
5. Family-level: protein kinase family and group (from ProKinO)
6. Pathway-level: reaction, pathway (from Reactome [58], last accessed on May 15th, 2018), and biological process (from AmiGO [59], last accessed on May 15th, 2018)
7. Sample-level: microsatellite instability, average ploidy, age, cancer originated tissue type, and histological classification (from COSMIC and Cellosaurus, [60]).

The formulas for generating all cancer cell line features are shown in Additional file 1: Table S9.

### QSMART model

The Quantitative Structure–Mutation–Activity Relationship Tests (QSMART) model was developed based on the QSAR model. First, we built a basic model with all drug features and cancer cell line features as independent variables for estimating $\hbox {IC}_{50}$:

$$\begin{aligned} IC_{50} = \beta _{0} + \sum _{i=1}^{I}\beta _{1i}D_{i} + \sum _{j=1}^{J}\beta _{2j}C_{j} + \epsilon , \end{aligned}$$

where $\beta _0$ is the intercept, $\beta _{1i}$ and $\beta _{2j}$ are the coefficients of the *i*th drug feature $D_i$ and the *j*th cancer cell line feature $C_j$, and $\epsilon$ is the error term.

Because the residue-level features of a cancer cell line represent the mutation status in the reference PKA structure, and we are interested in investigating drug–mutation relationships, we introduced drug–mutation interaction terms in the model:

$$\begin{aligned} IC_{50} = \beta _{0}+ \sum _{i=1}^{I}\beta _{1i}D_{i}+ \sum _{j=1}^{J}\beta _{2j}C_{j} + \sum _{i=1}^{I}\sum _{k=1}^{K}\beta _{3ik}D_{i}M_{k} + \epsilon , \end{aligned}$$

where $\beta _{3ik}$ is the coefficient of the interaction term formed by the *i*th drug feature $D_i$ and the *k*th residue-level feature $M_k$. Since all cancer cell line features contain residue-level features and the other six feature categories, $\{C_1, \ldots , C_J\}$ is a superset of $\{M_1, \ldots , M_K\}$. Considering that the interaction terms formed by the substructures of drug and high-level cancer cell line features have no biological relevance, we did not incorporate all cancer cell line features as part of interaction terms. For example, we did not consider the interaction between a substructure “Fingerprint_1” and a biological process “lung development” because it is unexplainable.

In addition to using all cancer cell line features, we further introduced additional interaction terms to capture various proteomic, cellular, and genomic features:

$$\begin{aligned} \begin{aligned} IC_{50}&= \beta _{0} + \sum _{i=1}^{I}\beta _{1i}D_{i} + \sum _{j=1}^{J}\beta _{2j}C_{j} + \sum _{i=1}^{I}\sum _{k=1}^{K}\beta _{3ik}D_{i}M_{k} \\&\quad +\,\sum _{p=1}^{P}\beta _{4p}PPI_{p} + \sum _{q=1}^{Q}\beta _{5q}RECx_{q} + \sum _{r=1}^{R}\beta _{6r}PWYx_{r} + \sum _{s=1}^{S}\beta _{7s}GOx_{s} + \epsilon , \end{aligned} \end{aligned}$$

where $\beta _{4p}$, $\beta _{5q}$, $\beta _{6r}$, and $\beta _{7s}$ are the coefficients of the *p*th protein–protein interaction $PPI_p$, the *q*th reaction–reaction interaction $RECx_q$, the *r*th pathway–pathway interaction $PWYx_r$, and the *s*th biological process interaction $GOx_s$, respectively. More details about interaction terms are described below.

### Interaction terms

Five types of interaction terms were introduced into the QSMART model: drug–mutation interaction, protein–protein interaction, reaction–reaction interaction, pathway–pathway interaction, and biological process interaction. These interactions were not necessarily physical; instead, they were predictors that show statistically significant contribution to explaining the variation of $\hbox {IC}_{50}$ values. For drug–mutation interaction terms, only the residue mapping to the reference PKA structure was considered to form interactions with drugs. For protein–protein interaction (PPI), we retained the non-self-interaction PPIs formed by at least one human protein kinase with interaction scores greater than 700 in the STRING database [61]. Gene expression level was used as a weight for PPIs to represent protein levels in cancer cell lines. For reaction, pathway, and biological process interactions, we removed the interactions formed by two entities from the same biological process/pathway hierarchy. For instance, the interaction between the biological process “lung cell differentiation” (GO:0060479) and its parent “lung development” (GO:0030324) was removed since it is unexplainable. Each interaction term was tested individually by F-test using R [62] (version 3.4.4). Significant interaction terms (FDR < 0.05) with no less than 30 non-zero values were used for further feature selection.

### Datasets

To reduce potential sources of noise and bias, we further filtered cancer cell lines from the PKI response dataset if (1) their mutation profiles are not detected by whole-genome sequencing, (2) they have less than 30 drug response entries, (3) their gene expression profile is not available, or (4) their mutation site does not map to a residue in the reference PKA position. The dataset was then split into 29 groups, stratified by primary cancer sites. Groups with less than 1000 responses (adrenal gland, biliary tract, placenta, prostate, salivary gland, small intestine, testis, and vulva) were excluded due to low statistical power. “Haematopoietic and lymphoid tissue”, the largest group, was further divided into two subsets by primary histology: “haematopoietic neoplasm” and “lymphoid neoplasm”. For the case study, we collected cancer cell lines for the non-small cell lung cancer (NSCLC) dataset from the lung cancer dataset if their histology subtype is adenocarcinoma, non-small cell carcinoma, squamous cell carcinoma, large cell carcinoma, giant cell carcinoma, or mixed adenosquamous carcinoma. Remaining lung cancer cell samples were classified as “lung (others)”. We created cancer type-centric training sets by expanding the drug response dataset with drug features, cancer cell lines features, and significant interaction terms. Categorical data in the training sets were coded into dummy variables. As a result, we prepared 23 cancer type-centric training sets. The number of PKI responses for each cancer type is shown in Table 2.

### Feature screening

Observing high multicollinearity within the features in the first component of our prediction framework (Fig. 1), we implemented the variance inflation factor criterion (VIF) [53] to remove highly correlated features. For the multiple regression model with *f* features, $X_i$ $(i=1, \ldots , f)$, the VIF for the *i*th feature can be expressed by: $VIF_{i}=\frac{1}{1-R^2_i}$, where $R^2_i$ is the coefficient of determination of the regression between $X_i$ and the remaining $f-1$ features. $VIF_i > 5$ (i.e. $R^2_i > 0.8$) is considered to be high collinearity and $X_i$ should be excluded from the model [53]. We first prioritized drug features based on these rules: (1) the later PubChem fingerprint bit positions (complex patterns) have higher priorities than the earlier ones (simple elements), and (2) PubChem fingerprints have higher priorities than calculated chemical descriptors because fingerprints directly represent molecular substructures of the drug. Chemical descriptors, such as ALogP [63], are calculated or estimated based on multiple substructures. In our study, because we considered the interactions between these high-level drug features and mutations were not easily explainable, we chose to assign low priorities to these drug features when performing feature screening. This process can be viewed as feature engineering based on domain knowledge [64]. Essentially, if experts understand what the features mean, they will better interpret the model. Then, we implemented stepwise selection (starting from higher priority features) under VIF control. Co-expressed genes in the same prediction model also exhibited collinearity. To address this issue, we also used the VIF criterion to filter co-expressed genes in each training set.

### Feature selection

To combat the problem of p (the number of drug features plus cancer cell line features and interaction terms) $>>$ n (the number of drug responses) in the training sets, we implemented Lasso [65] with Bayesian Information Criterion (BIC) [66] by an R package “HDeconometrics” [67]. Lasso is appropriate for estimating coefficients in high-dimensional space [68], while BIC provides an efficient approach to select the optimal Lasso model [69]. Under the condition of a fixed number of drug responses, the model was penalized based on the number of selected features when minimizing BIC:

$$\begin{aligned} BIC = k\cdot ln(n)-2ln({\widehat{L}}), \end{aligned}$$

where ${\widehat{L}}$ is the maximum likelihood of the model, k is the number of features in the model, and n is the number of observations (drug responses) used in the model. After feature selection, the remaining number of selected features for each cancer type is shown in Table 2.

Additionally, we performed three distinct feature selection methods with different underlying assumptions and one ensemble method. We used WEKA’s correlation attribute evaluation, ReliefF, and classifier (random forests) attribute evaluation to rank features [70], and then calculated each feature’s average rank in Lasso and these three methods. To make the results comparable, we selected the same number of features as those we selected using Lasso under BIC control.

### Neural network architecture

We built neural network models by using $\hbox {JMP}^{\textregistered }$ [71]. We designed three types of neural network architectures in this study: single-layer, double-layer, and complex-double-layer. The numbers of hidden layer nodes follow the geometric pyramid rule [72]. Given N input nodes objectively determined by the feature selection methods, there are $\lceil N^{1/2} \rceil$ hidden nodes in a single-layer architecture. In a double-layer architecture, there are $\lceil N^{2/3} \rceil$ and $\lceil N^{1/3} \rceil$ hidden nodes in the first and second hidden layers, respectively. In a complex-double-layer architecture, there are N and $\lceil N^{1/2} \rceil$ hidden nodes in the first and second hidden layers, respectively. The nodes among the two layers are fully connected. Biases are introduced into the input and hidden layers. The activation function of every node is a hyperbolic tangent function (TanH). A quasi-Newton method, BFGS [73], is chosen as an optimizer by JMP.

To mitigate overfitting, we performed 10-fold cross-validation, early stopping, and Lasso-style penalty function (absolute value penalty, i.e., $\hbox {L}_{1}$ regularization [74]). When performing 10-fold cross-validation, we partitioned the observations (drug responses) into ten folds. In turn, each fold served as a validation set to evaluate the model built upon the rest nine folds. The tuning parameters that construct the model giving the best validation statistics were selected in the final model. The average performance ($\hbox {R}^{2}$) of the ten models for each cancer type was reported. To tune the hyperparameters, we started from a single-layer model for each cancer type based on Occam’s razor principle [75]. If the performance is less than the threshold of 0.8 in 200 iterations, we increased the number of iterations to 300; if the performance is still less than the threshold, we implemented a double-layer model for 200 iterations, and so on until using a complex-double-layer model for 300 iterations.

### Other machine learning and drug responses prediction methods

We compared neural networks with three other prediction algorithms with 10-fold cross-validation: random forests, support vector machine (SVM), and elastic net. Random forests were implemented by WEKA [70] (version 3.8.3). For each cancer type, the number of iterations was decided based on the iterations used for each of the pre-trained neural network models (200 or 300 iterations) shown in Additional file 1: Table S1. SVM was implemented by the SMOreg function (SVM for regression) of WEKA. Elastic net was implemented by an R package “glmnet” [76]. To optimize the parameter settings for the compared machine learning methods, we used the grid search method. We built 100 models with different parameter combinations for each method. Detailed parameter values are available in Additional file 5.

Additionally, we also compared our prediction models with two-way ANOVA analysis and a drug response prediction model, multiscale convolutional attentive (MCA) [36]. Because the purpose of two-way ANOVA analysis implemented by R was to quantify how much two factors (drug and cancer cell line) can explain the variation of drug response (adjusted $\hbox {R}^{2}$ was used), the model used the drug and cancer cell line identifiers as inputs and did not undergo 10-fold cross-validation. MCA combines gene expression profiles, the molecular structure of compounds, and prior knowledge of protein-protein interactions, and uses convolutional neural networks to predict drug response. The performance of MCA for PKI response prediction is available in Additional file 2.

## Supplementary information

**Additional file 1.** Supplementary results, figures, and tables.

**Additional file 2.** MCA’s performance for PKI response prediction.

**Additional file 3.** Selected features in NSCLC dataset.

**Additional file 4.** PKI target groups and PKI structures.

**Additional file 5.** Prediction performances.

## Data Availability

Training sets, the codes for building prediction models, and prediction results are available at https://github.com/esbgkannan/QSMART/.

## Abbreviations

ANOVA: Analysis of variance
AUC: Area under the receiver operating characteristic curve
BIC: Bayesian information criterion
CCLE: Cancer Cell Line Encyclopedia
DNN: Deep neural networks
GDSC: Genomics of Drug Sensitivity in Cancer
MCA: Multiscale convolutional attentive
NSCLC: Non-small cell lung cancer
PKA: Protein kinase A
PKI: Protein kinase inhibitor
PPI: Protein–protein interaction
RMSE: Root-mean-square error
QSAR: Quantitative structure–activity relationship
QSMART: Quantitative structure–mutation–activity relationship tests
SVM: Support vector machine
VIF: Variance inflation factor
XAI: Explainable artificial intelligence
