# Supplementary material for: Quantitative Structure–Mutation–Activity Relationship Tests (QSMART) model for protein kinase inhibitor response prediction
Source: BMC Bioinformatics. 2020 Nov 12;21:520. doi: 10.1186/s12859-020-03842-6 (PMC7664030; doi:10.1186/s12859-020-03842-6)
Supplement: Supplementary file 1 — Additional file 1. Supplementary results, figures, and tables. [file 12859_2020_3842_MOESM1_ESM.pdf]

RESEARCH ARTICLE

Quantitative Structure-Mutation-Activity Relationship Tests (QSMART) Model for Protein Kinase Inhibitor Response Prediction

Liang-Chin Huang[1], Wayland Yeung[1], Ye Wang[2], Huimin Cheng[2], Aarya Venkat[3], Sheng Li[4], Ping Ma[2], Khaled Rasheed[4] and Natarajan Kannan[1,3*]

Supplementary Results

Residual analysis

Residual analysis was then performed to assess the appropriateness of our prediction models. The residual plots for all the 23 cancer types (Figure S3) show no specific U shape, inverted U shape, or funnel shape, which means our prediction models need no more higher-order features to capture drug response variation.

Analysis of the feature prioritization in feature screening

Considering the explainabilities of drug features, we prioritized them when we performed feature screening. Because it was a drug response-independent preprocess, the screening result (92 fingerprints and no chemical descriptors) did not indicate that chemical descriptors were not informative for drug response prediction. Instead, it was the consequence of the high collinearity among drug features and our prioritization. We reversed our prioritization and performed an additional experiment to investigate the result of feature screening. As a result, four fingerprints and 29 chemical descriptors remained (Table S10). Since high multicollinearity exists among the raw drug features, either screening result can be representative of the raw drug features, but the 92 fingerprints illuminate the "black box" between feature and response.

Contribution of different feature categories

To roughly estimate the contribution of different feature types to the prediction accuracy, we split the features into three categories: drug features, cancer cell line features, and interaction terms. We used the same neural network architecture (the number of nodes in the first and second hidden layers) in each cancer-centric model, and then built prediction models using the split feature sets. Across the 23 cancer types, this experiment showed that using drug features alone to predict PKI response outperformed using cancer cell line features or interaction terms alone (overall $R^2 =$ 0.661, 0.126, and 0.152, respectively; Table S11). The contribution of interaction terms to prediction performance was significant (p-value = 0.0041, Wilcoxon signed-rank test) in comparison to cancer cell line features. Although it was partially due to the number of selected drug features being more than those of the other two feature categories, the main reason was that the drug features were more informative in cancer-centric models. Since the entire training dataset was split into 23 cancer-centric datasets, the similarity among cancer cell lines in one dataset was higher than the similarity among PKIs. Thus, the drug features had higher variation and higher entropy.

Assuming that the features from different categories in a full model are independent and can explain the variation of drug response independently, the summation of the prediction performances of split models (the R^2_{SSP} in Table S11) would ideally be the upper limit of a full model. However, Table S11 shows that the prediction performances R^2_{Full} are even higher than R^2_{SSP} for 14 cancer types, which implies that the synergistic prediction performances (R^2_{Full} - R^2_{SSP}) are potentially derived from the higher-order interactions performed by neural networks. Interestingly, we found that the neural network architectures with the top four synergistic effects are double-layer neural networks instead of single-layer neural networks. It supports our hypothesis that synergistic prediction performance is derived from higher-order interactions.

Prediction performance for different PKI target groups

Some previous studies [1, 2] built both drug-centric and cancer-centric models to predict drug response. However, since our study focused on investigating drug-mutation relationships, we did not build drug-centric models. If we apply the framework in our study to a single drug, all the drug features will be the same

*Correspondence: nkannan@uga.edu
[1] Institute of Bioinformatics, University of Georgia, 120 Green St., 30602, Athens, GA, USA
[3] Department of Biochemistry and Molecular Biology, 120 Green St., 30602, Athens, GA, USA
Full list of author information is available at the end of the article

across different drug response samples. Thus no significant drug features nor significant interaction terms will be captured. Nevertheless, we were still interested in the prediction performances for different drugs. The prediction model having the best validation performance across the 10-fold cross-validation was chosen as the final model for each cancer type. We used the model to predict drug response for the entire training set and then gathered prediction results for each PKI. We pooled the results of PKIs according to their target groups (Additional file 4) into nine sets: AGC, CAMK, CK1, CMGC, STE, TK, TKL, Other, and Atypical. One PKI response prediction might be pooled in one or multiple sets since one PKI may have one or more drug targets classified as different protein kinase groups. We first analyzed the average actual IC_{50} in different PKI target group sets. The result showed that if a drug inhibits CMGC, CAMK, or AGC protein kinases, it has higher average IC_{50} values for most cancer types (average IC_{50} = 2.527, 2.389, and 2.331, respectively. Figure S4a). Contrarily, if a drug inhibits Atypical and CK1 protein kinases, it has lower average IC_{50} values (1.624 and 1.679, respectively). This result was according to the data we collected from GDSC, and it might not be applied to all the cases. Figure S4b shows the detailed performances evaluated by R^2. To our surprise, we found the best is Atypical group (R^2 = 0.699 to 0.901 and overall R^2 = 0.872) and the worst is CAMK group (R^2 = 0.644 to 0.885 and overall R^2 = 0.785).

Although atypical protein kinases lack canonical protein kinase domains, the models could still predict atypical protein kinase inhibitor responses well. We speculated that the performance was supported by independent drug features, cancer cell line features, or the drug-mutation relationships from unknown off-targets. The mammalian target of rapamycin (mTOR), classified as Atypical group, is another potential factor to explain this result. mTOR regulates cell growth, proliferation, motility, and survival [3], and it is highly mutated in the cancer cell lines in our dataset: 67 out of 837 cell lines (8%) have mTOR mutations. Since it is critical to cell activity, the six drugs that inhibit mTOR (listed in Additional file 4) might require less concentration to inhibit the cancer cell line's activity. Moreover, since mTOR is implicated in a broad category of pathways, each of its mutations provides more information about the sample's cancer cell line features to the prediction models. On the contrary, although the CAMK group proteins have canonical protein kinase domains, the models could not predict CAMK inhibitor responses well. We conjectured that this was because none of the PKIs in our dataset specifically inhibit CAMK group proteins so that the models were not tailored to capture CAMK inhibitor-specific features and interaction terms. Although there are 33 CAMK inhibitors in our dataset (Additional file 4), all of them had at least one more target classified as other groups. Compared to CAMK inhibitors, atypical PKIs had relatively higher specificity in this point of view. There are 29 atypical PKIs in our dataset, and 8 of them (27.6%) only inhibit their targets classified as Atypical group.

More explanations about the features in the case study

In addition to the features explained in the main article, we choose more features and explain their biological relevance to the NSCLC case study.

Gene-level feature

"CNV_ROCK2_gain". This feature represents if Rho-associated protein kinase 2 (ROCK2) is either neutral or deleted in a cancer cell line (0, copy number losses) or amplified (1, copy number gains). ROCK2 is known to be essential for NSCLC's growth and invasion [4]. In the NSCLC dataset, ROCK2 is amplified in two cell lines: LC-1/sq and NCI-H1623; the latter's source was from a patient with metastatic NSCLC. On average, the PKI responses involved in the cell lines with neutral or deleted ROCK2 showed lower IC_{50} value than those with amplified ROCK2 (average actual IC_{50} = 2.71 vs. 3.49). By using the pre-trained model, however, when the value of CNV_ROCK2_gain was replaced from 0 to 1 when other features were held constant, the estimated IC_{50} decreased 0.14 on average (average predicted IC_{50} = 2.71 vs. 2.57). Although the coefficient of CNV_ROCK2_gain obtained from Lasso feature selection was 0.07, meaning it positively correlated to IC_{50}, the neural network model had not perfectly learned this trend.

Pathway-level feature

"REC_R_HSA_176298". This feature shows the number of mutations in the proteins implicating in the reaction "Activation of claspin" (Reactome ID: R-HSA-176298). Claspin is an essential regulator for checkpoint kinase 1 (Chk1) activation, and it was found to be associated with regulating breast cancer proliferation [5, 6] and contributing to lung cancer radioresistance [7]. Interestingly, this feature was also selected in our PKI response prediction model for breast cancer cell lines. On average, the NSCLC cell lines without mutations related to claspin activation had lower PKI responses than those with mutations related to this reaction (average actual IC_{50} = 2.66 vs. 3.27). Based on the pre-trained neural network model and our NSCLC dataset, every unit increase in REC_R_HSA_176298 is associated with a 0.52 unit increase in IC_{50} on average (average predicted IC_{50} = 2.73 vs. 3.25).

Understudied protein-protein interaction

CDK13, an understudied protein kinase defined by NIH Illuminating the Druggable Genome program (IDG) [8] (Additional file 5, last updated on June 11, 2019), participates in a 4-clique PPI module in the TP53-centric subnetwork (Figure 3). Its three PPIs in this module are all the features of the NSCLC-specific model. One of CDK13's PPI partners, AKAP4, is a biomarker for NSCLC, and its expression increase was associated with tumor stage [9]. In addition to NSCLC, AKAP4 is also a potential therapeutic target of colorectal cancer [10] and ovarian cancer [11], and it regulates the expression of the CDK family. In the NSCLC dataset, the expression of CDK13-AKAP4 interaction had a weak positive correlation with IC_{50} (Pearson correlation = 0.07); in the prediction model, every unit of gene expression level increase in CDK13-AKAP4 PPI is associated with a 0.017 unit increase in IC_{50} on average (average predicted IC_{50} = 2.727 vs. 2.744).

Author details

[1] Institute of Bioinformatics, University of Georgia, 120 Green St., 30602, Athens, GA, USA. [2] Department of Statistics, University of Georgia, 310 Herty Drive, 30602, Athens, GA, USA. [3] Department of Biochemistry and Molecular Biology, 120 Green St., 30602, Athens, GA, USA. [4] Department of Computer Science, 415 Boyd Graduate Studies Research Center, 30602, Athens, GA, USA.

References

1. Chang, Y., Park, H., Yang, H.J., Lee, S., Lee, K.Y., Kim, T.S., Jung, J., Shin, J.M.: Cancer Drug Response Profile scan (CDRscan): A Deep Learning Model That Predicts Drug Effectiveness from Cancer Genomic Signature. Sci Rep **8**(1), 8857 (2018)
2. Liu, H., Zhao, Y., Zhang, L., Chen, X.: Anti-cancer Drug Response Prediction Using Neighbor-Based Collaborative Filtering with Global Effect Removal. Mol Ther Nucleic Acids **13**, 303–311 (2018)
3. Hay, N., Sonenberg, N.: Upstream and downstream of mTOR. Genes Dev. **18**(16), 1926–1945 (2004)
4. Vigil, D., Kim, T.Y., Plachco, A., Garton, A.J., Castaldo, L., Pachter, J.A., Dong, H., Chen, X., Tokar, B., Campbell, S.L., Der, C.J.: ROCK1 and ROCK2 are required for non-small cell lung cancer anchorage-independent growth and invasion. Cancer Res. **72**(20), 5338–5347 (2012)
5. Lin, S.Y., Li, K., Stewart, G.S., Elledge, S.J.: Human Claspin works with BRCA1 to both positively and negatively regulate cell proliferation. Proc. Natl. Acad. Sci. U.S.A. **101**(17), 6484–6489 (2004)
6. Verlinden, L., Vanden Bempt, I., Eelen, G., Drijkoningen, M., Verlinden, I., Marchal, K., De Wolf-Peeters, C., Christiaens, M.R., Michiels, L., Bouillon, R., Verstuyf, A.: The E2F-regulated gene Chk1 is highly expressed in triple-negative estrogen receptor /progesterone receptor /HER-2 breast carcinomas. Cancer Res. **67**(14), 6574–6581 (2007)
7. Choi, S.H., Yang, H., Lee, S.H., Ki, J.H., Nam, D.H., Yoo, H.Y.: TopBP1 and Claspin contribute to the radioresistance of lung cancer brain metastases. Mol. Cancer **13**, 211 (2014)
8. the Druggable Genome, I.: Understudied Proteins. https://commonfund.nih.gov/idg/understudiedproteins. Accessed: 2019-06-11 (2019)
9. Gumireddy, K., Li, A., Chang, D.H., Liu, Q., Kossenkov, A.V., Yan, J., Korst, R.J., Nam, B.T., Xu, H., Zhang, L., Ganepola, G.A., Showe, L.C., Huang, Q.: AKAP4 is a circulating biomarker for non-small cell lung cancer. Oncotarget **6**(19), 17637–17647 (2015)
10. Jagadish, N., Parashar, D., Gupta, N., Agarwal, S., Purohit, S., Kumar, V., Sharma, A., Fatima, R., Topno, A.P., Shaha, C., Suri, A.: A-kinase anchor protein 4 (AKAP4) a promising therapeutic target of colorectal cancer. J. Exp. Clin. Cancer Res. **34**, 142 (2015)
11. Kumar, V., Jagadish, N., Suri, A.: Role of A-Kinase anchor protein (AKAP4) in growth and survival of ovarian cancer cells. Oncotarget **8**(32), 53124–53136 (2017)

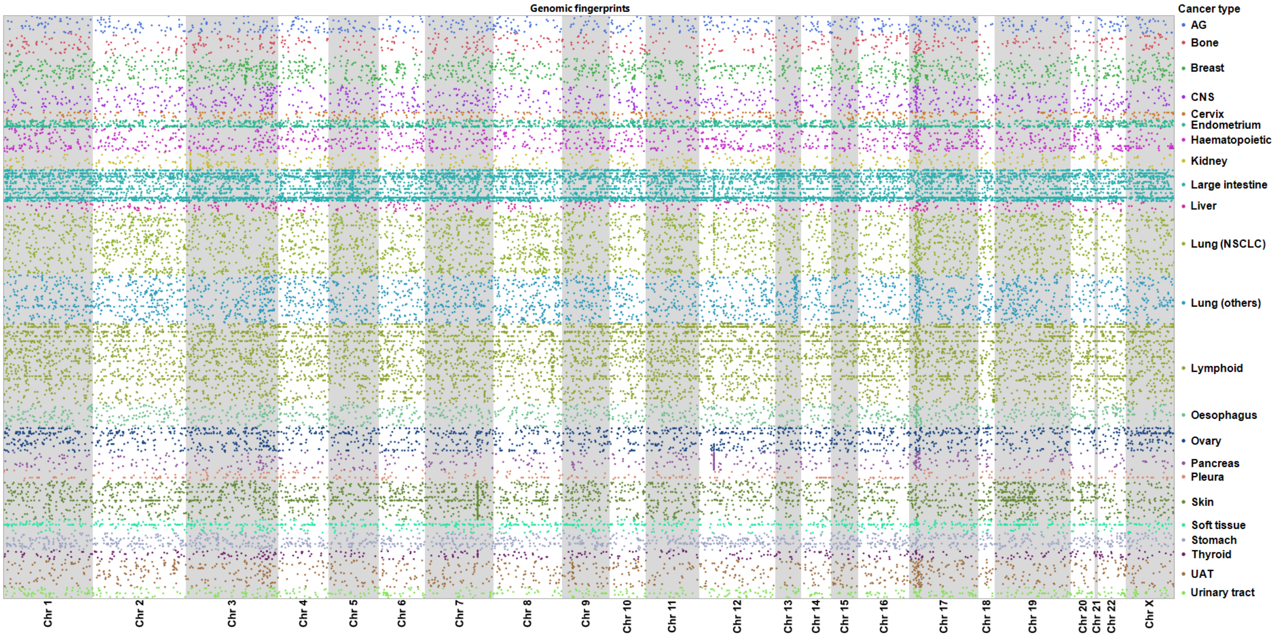

Figure S1 Genome-wide mutational status (genomic fingerprints) across the 23 cancer types. AG: autonomic ganglia; CNS: central nervous system; NSCLC: non-small cell lung cancer; UAT: upper aerodigestive tract.

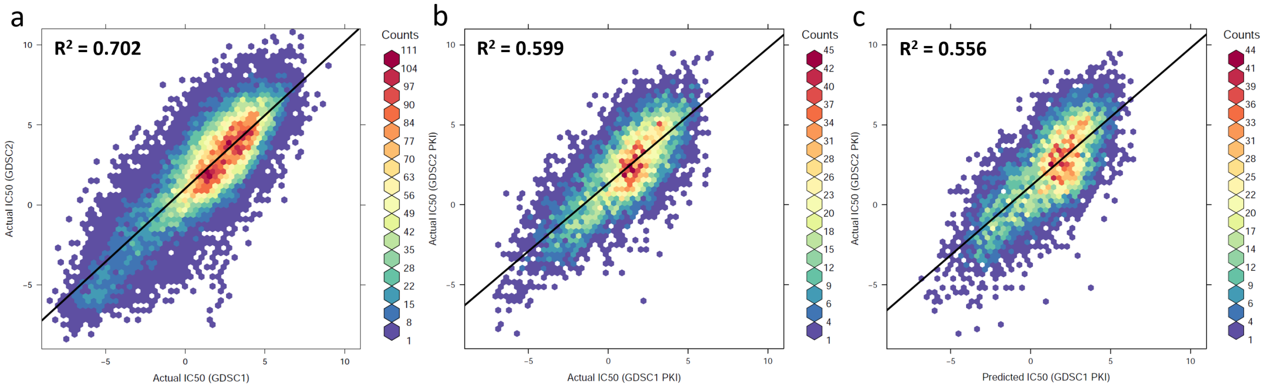

Figure S2 Comparison between GDSC1 and GDSC2. GDSC1 (the old drug response dataset in GDSC release 8.0) and GDSC2 (the new drug response dataset in GDSC release 8.0) were generated based on different protocols. Cell viability was measured using either Resazurin or Syto60 in GDSC1, while it was measured based on Promega CellTiter-Glo® in GDSC2. In total, there are 22,624 drug-cancer cell line pairs found in both datasets; Wellcome Sanger Institute performed all the experiments. (a) The hexbin plot shows the actual IC_{50} from GDSC1 (x-axis) versus the actual IC_{50} from GDSC2 (y-axis). A fitted regression line and its R^2 are shown. (b) There are 7,283 PKI-cancer cell line pairs found in both GDSC1 and GDSC2. The hexbin plot shows the PKI's actual IC_{50} from GDSC1 (x-axis) versus the PKI's actual IC_{50} from GDSC2 (y-axis). (c) Based on the prediction result of our QSMART with neural network models trained by GDSC1 data, the hexbin plot shows the PKI's predicted IC_{50} (x-axis) versus the PKI's actual IC_{50} from GDSC2 (y-axis).

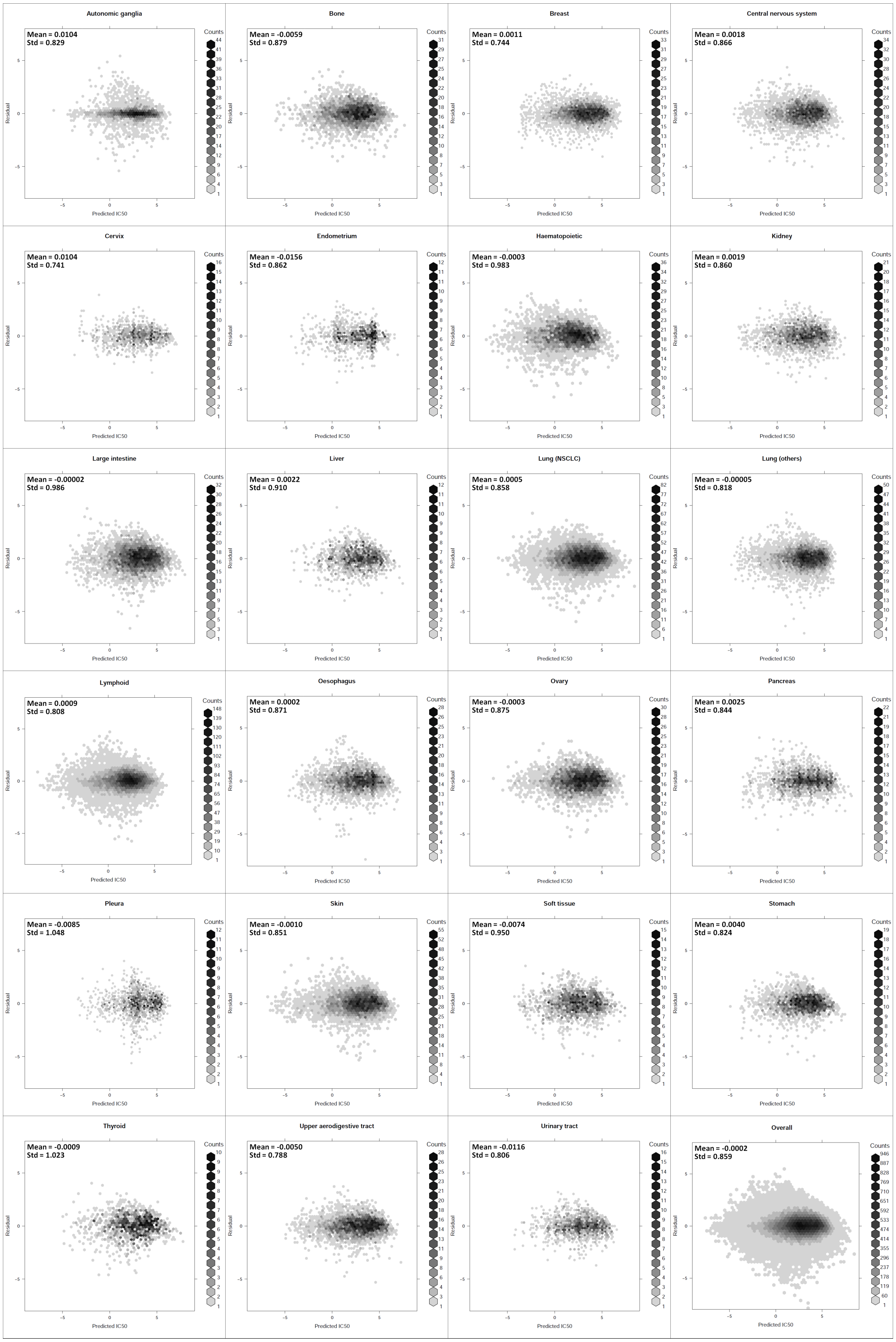

Figure S3 Residual analyses for 23 cancer-centric models and the overall result. X-axis: predicted IC_{50}; y-axis: residuals, defined as actual IC_{50} minus predicted IC_{50}. Residuals mean and standard deviation are shown for each cancer type.

a Average IC_{50}

PKI target group

	AGC	CAMK	CK1	CMGC	STE	TK	TKL	Other	Atypical
Autonomic ganglia	2.104	2.256	1.848	2.495	2.006	2.123	1.984	1.941	1.469
Bone	2.036	2.120	1.389	2.249	1.887	1.914	1.786	1.727	1.296
Breast	2.953	3.027	2.156	3.081	2.714	2.721	2.692	2.761	2.316
Central nervous system	2.434	2.486	1.901	2.658	2.187	2.339	2.178	2.282	1.802
Cervix	3.120	3.068	2.384	3.231	2.709	2.644	2.964	2.852	2.181
Endometrium	2.479	2.585	1.496	2.681	2.285	2.374	2.259	2.296	1.855
Haematopoietic	1.098	1.181	0.694	1.344	0.880	1.049	0.976	0.816	0.226
Kidney	2.410	2.612	2.061	2.770	2.238	2.327	2.284	2.403	1.825
Large intestine	2.820	2.868	2.133	3.008	2.355	2.601	2.624	2.571	2.192
Liver	2.844	2.910	2.495	2.999	2.461	2.452	2.707	2.634	2.045
Lung (NSCLC)	2.806	2.827	2.070	2.893	2.461	2.526	2.609	2.541	2.128
Lung (others)	2.589	2.676	1.905	2.818	2.485	2.488	2.499	2.299	1.951
Lymphoid	1.301	1.441	0.726	1.565	1.285	1.458	1.263	1.069	0.492
Oesophagus	2.480	2.589	1.755	2.702	2.216	2.285	2.379	2.335	1.919
Ovary	2.806	2.679	1.909	2.851	2.225	2.473	2.462	2.446	1.935
Pancreas	3.051	3.034	2.272	3.050	2.483	2.657	2.801	2.667	2.257
Pleura	2.976	3.050	2.619	3.168	2.768	2.782	2.820	2.911	2.338
Skin	2.419	2.426	1.724	2.686	1.853	2.311	2.025	2.285	1.680
Soft tissue	2.204	2.293	1.607	2.455	2.001	2.126	1.990	2.043	1.365
Stomach	2.605	2.707	1.857	2.750	2.297	2.408	2.446	2.437	1.821
Thyroid	2.482	2.497	2.009	2.662	2.024	2.267	2.141	2.397	1.716
Upper aerodigestive tract	2.744	2.608	2.005	2.771	2.198	2.330	2.511	2.435	1.914
Urinary tract	2.833	2.776	2.307	2.869	2.410	2.469	2.601	2.544	2.048
Overall	2.331	2.389	1.679	2.527	2.055	2.199	2.144	2.105	1.624

Max / Min

b Overall R^2

PKI target group

	AGC	CAMK	CK1	CMGC	STE	TK	TKL	Other	Atypical
Autonomic ganglia	0.824	0.735	0.896	0.846	0.826	0.798	0.850	0.830	0.887
Bone	0.835	0.775	0.850	0.846	0.797	0.810	0.811	0.826	0.856
Breast	0.874	0.805	0.888	0.870	0.861	0.854	0.845	0.867	0.893
Central nervous system	0.817	0.710	0.856	0.823	0.777	0.802	0.785	0.799	0.860
Cervix	0.891	0.885	0.927	0.892	0.891	0.868	0.859	0.898	0.900
Endometrium	0.842	0.806	0.892	0.867	0.829	0.815	0.857	0.826	0.895
Haematopoietic	0.837	0.804	0.872	0.846	0.803	0.828	0.827	0.827	0.847
Kidney	0.848	0.770	0.901	0.856	0.824	0.822	0.853	0.828	0.891
Large intestine	0.773	0.644	0.732	0.763	0.771	0.746	0.739	0.757	0.785
Liver	0.803	0.708	0.831	0.785	0.841	0.808	0.780	0.829	0.820
Lung (NSCLC)	0.821	0.741	0.840	0.825	0.821	0.817	0.799	0.820	0.857
Lung (others)	0.841	0.776	0.876	0.845	0.824	0.817	0.817	0.833	0.871
Lymphoid	0.857	0.812	0.874	0.869	0.823	0.837	0.830	0.841	0.873
Oesophagus	0.829	0.689	0.841	0.846	0.785	0.807	0.825	0.800	0.873
Ovary	0.825	0.784	0.865	0.822	0.857	0.815	0.811	0.848	0.856
Pancreas	0.837	0.750	0.825	0.814	0.855	0.840	0.806	0.863	0.843
Pleura	0.670	0.709	0.794	0.691	0.699	0.680	0.769	0.642	0.699
Skin	0.841	0.772	0.834	0.844	0.868	0.826	0.837	0.823	0.871
Soft tissue	0.840	0.732	0.877	0.821	0.786	0.795	0.810	0.800	0.878
Stomach	0.832	0.756	0.850	0.835	0.815	0.818	0.811	0.811	0.886
Thyroid	0.822	0.756	0.806	0.801	0.824	0.762	0.776	0.801	0.809
Upper aerodigestive tract	0.853	0.824	0.887	0.859	0.883	0.856	0.845	0.869	0.901
Urinary tract	0.839	0.764	0.850	0.832	0.859	0.824	0.813	0.833	0.859
Overall	0.846	0.785	0.865	0.850	0.833	0.826	0.830	0.838	0.872

Max / Min

Figure S4 Prediction performances of using QSMART model with neural networks for different PKI target groups. (a) Average actual IC_{50} of different PKI target groups across 23 cancer types. (b) The prediction performances (R^2) of using QSMART model with neural networks for different PKI target groups. NSCLC: non-small cell lung cancer.

Table S1 Number of features at different feature levels and the prediction performance of neural networks

Cancer type	#IC_{50}	#All Features	#Drug Features	#Cancer cell line features							#Interaction terms					#Nodes		#Tours	Performance		
				Residue	Motif	Domain	Gene	Family	Pathway	Sample	DxM	PPI	RECx	PWYx	GOx	1st	2nd		R^2	RMSE	AUC
AG	2971	62	31	0	0	0	6	3	0	0	4	18	0	0	0	62	8	200	0.879	0.688	0.978
Bone	3410	84	52	0	1	0	1	0	11	0	4	11	0	3	1	10	0	300	0.856	0.812	0.984
Breast	4706	129	70	5	0	1	10	0	15	0	12	6	1	5	4	26	6	200	0.880	0.714	0.986
CNS	4250	114	65	0	0	0	9	1	12	1	11	6	1	4	4	11	0	300	0.858	0.785	0.980
Cervix	1044	37	29	0	0	0	2	0	1	0	1	4	0	0	0	7	0	200	0.864	0.770	0.989
Endometrium	1073	33	21	0	0	0	0	0	3	1	4	3	0	0	1	11	4	200	0.878	0.733	0.982
Haematopoietic	4204	119	58	3	0	2	9	0	13	0	28	2	0	0	4	11	0	200	0.858	0.906	0.971
Kidney	2458	73	51	0	0	0	1	0	1	1	0	17	1	0	1	9	0	200	0.836	0.877	0.986
Large intestine	4628	141	53	10	1	1	4	0	8	0	50	10	1	3	0	12	0	300	0.814	0.923	0.974
Liver	1348	48	35	0	0	0	0	2	2	0	2	6	0	0	1	7	0	200	0.836	0.844	0.985
Lung (NSCLC)	9205	207	72	7	0	0	9	4	21	1	47	27	1	3	15	15	0	200	0.854	0.809	0.982
Lung (others)	7206	162	58	2	0	0	3	1	11	1	46	23	0	4	13	30	6	200	0.859	0.756	0.983
Lymphoid	13302	291	72	54	0	2	11	1	14	2	86	39	4	0	6	18	0	300	0.873	0.757	0.980
Oesophagus	3337	91	58	0	0	0	8	0	9	0	4	9	0	1	2	10	0	200	0.841	0.857	0.972
Ovary	3502	113	64	2	0	1	9	3	5	0	9	17	1	0	2	11	0	200	0.844	0.867	0.987
Pancreas	2421	84	60	0	0	1	2	1	3	0	0	13	0	3	1	10	0	200	0.833	0.877	0.990
Pleura	1431	36	23	0	1	2	2	0	0	0	0	8	0	0	0	11	4	200	0.805	0.894	0.966
Skin	5732	132	64	9	0	1	7	0	13	0	15	15	0	3	5	12	0	200	0.875	0.810	0.987
Soft tissue	1938	63	45	0	1	0	1	1	7	0	2	5	0	1	0	8	0	200	0.818	0.941	0.975
Stomach	2327	83	49	0	0	0	8	1	4	0	16	5	0	0	0	20	5	200	0.836	0.837	0.981
Thyroid	1352	33	25	0	0	0	5	0	0	0	0	2	0	1	0	6	0	300	0.830	0.963	0.973
UAT	3856	126	50	1	1	0	6	1	6	0	4	44	0	0	13	12	0	300	0.881	0.760	0.989
Urinary tract	1454	68	47	0	0	0	3	0	2	0	9	6	0	0	1	9	0	200	0.863	0.750	0.988
Overall	87155																		0.863	0.811	0.981

AG: autonomic ganglia; AUC: area under the ROC Curve; CNS: central nervous system; DxM: drug-mutation interaction term; GOx: biological process interaction; NSCLC: non-small cell lung cancer; PPI: protein-protein interaction; PWYx: pathway-pathway interaction; R^2: coefficient of determination; RECx: reaction-reaction interaction; RMSE: root-mean-square error; UAT: upper aerodigestive tract; #IC_{50}: the number of drug responses; #Nodes: the number of nodes in the first and second hidden layers of neural networks; #Tours: the number of times to restart the fitting process.

Table S2 Prediction performances of using genomic fingerprints

Cancer type	$\#IC_{50}$	#All Features	#Drug Features	#Genomics Fingerprints	#Interaction Terms	#Nodes		#Tours	Performance	
						1st	2nd		R^2	RMSE
AG	2971	62	29	0	33	62	8	200	0.613	1.235
Bone	3410	84	16	68	0	10	0	300	0.506	1.506
Breast	4706	129	25	98	6	26	6	200	0.648	1.221
CNS	4250	114	30	83	1	11	0	300	0.705	1.132
Cervix	1044	37	3	34	0	7	0	200	0.252	1.879
Endometrium	1073	33	3	30	0	11	4	200	0.252	1.858
Haematopoietic	4204	119	20	82	17	11	0	200	0.636	1.452
Kidney	2458	73	15	58	0	9	0	200	0.546	1.461
Large intestine	4628	141	24	111	6	12	0	300	0.648	1.269
Liver	1348	48	11	33	4	7	0	200	0.620	1.286
Lung (NSCLC)	9205	207	30	167	10	15	0	200	0.696	1.164
Lung (others)	7206	162	26	134	2	30	6	200	0.681	1.137
Lymphoid	13302	291	32	245	14	18	0	300	0.755	1.052
Oesophagus	3337	91	18	73	0	10	0	200	0.639	1.289
Ovary	3502	113	18	95	0	11	0	200	0.610	1.373
Pancreas	2421	84	12	72	0	10	0	200	0.415	1.643
Pleura	1431	36	5	31	0	11	4	200	0.253	1.738
Skin	5732	132	27	104	1	12	0	200	0.641	1.375
Soft tissue	1938	63	17	45	1	8	0	200	0.577	1.434
Stomach	2327	83	19	50	14	20	5	200	0.691	1.157
Thyroid	1352	33	8	24	1	6	0	300	0.488	1.672
UAT	3856	126	44	60	22	12	0	300	0.729	1.147
Urinary tract	1454	68	13	51	4	9	0	200	0.569	1.312
Overall	87155								0.655	1.289

AG: autonomic ganglia; CNS: central nervous system; NSCLC: non-small cell lung cancer; R^2: coefficient of determination; RMSE: root-mean-square error; UAT: upper aerodigestive tract; $\#IC_{50}$: the number of drug responses; #Nodes: the number of nodes in the first and second hidden layers of neural networks; #Tours: the number of times to restart the fitting process.

Table S3 Prediction performances of using no drug-mutation interaction terms

Cancer type	$\#IC_{50}$	#All Features	#Drug Features	#Cancer features		#Interaction terms				#Nodes		#Tours	Performance	
				Residue	Others	PPI	RECx	PWYx	GOx	1st	2nd		R^2	RMSE
AG	2971	62	36	0	11	15	0	0	0	62	8	200	0.851	0.766
Bone	3410	84	58	0	13	11	0	1	1	10	0	300	0.836	0.868
Breast	4706	129	74	3	31	6	1	9	5	26	6	200	0.880	0.712
CNS	4250	114	74	0	28	2	0	5	5	11	0	300	0.867	0.760
Cervix	1044	37	31	0	2	4	0	0	0	7	0	200	0.891	0.693
Endometrium	1073	33	25	0	5	3	0	0	0	11	4	200	0.807	0.925
Haematopoietic	4204	119	76	4	27	3	0	0	9	11	0	200	0.861	0.898
Kidney	2458	73	53	0	4	15	0	0	1	9	0	200	0.750	1.088
Large intestine	4628	141	76	20	25	11	7	2	0	12	0	300	0.837	0.863
Liver	1348	48	35	0	5	7	0	0	1	7	0	200	0.777	0.981
Lung (NSCLC)	9205	207	80	36	36	26	0	9	20	15	0	200	0.726	1.107
Lung (others)	7206	162	80	12	21	27	0	9	13	30	6	200	0.892	0.660
Lymphoid	13302	291	80	123	34	45	5	0	4	18	0	300	0.892	0.697
Oesophagus	3337	91	64	0	14	10	0	2	1	10	0	200	0.830	0.882
Ovary	3502	113	69	2	14	18	2	6	2	11	0	200	0.850	0.852
Pancreas	2421	84	60	0	7	13	0	3	1	10	0	200	0.839	0.862
Pleura	1431	36	25	0	4	7	0	0	0	11	4	200	0.701	1.104
Skin	5732	132	63	15	28	10	2	7	7	12	0	200	0.864	0.846
Soft tissue	1938	63	46	0	11	5	0	1	0	8	0	200	0.728	1.166
Stomach	2327	83	58	2	18	4	0	0	1	20	5	200	0.874	0.731
Thyroid	1352	33	25	0	5	2	0	1	0	6	0	300	0.653	1.362
UAT	3856	126	50	1	14	54	0	0	7	12	0	300	0.881	0.757
Urinary tract	1454	68	54	0	5	9	0	0	0	9	0	200	0.854	0.765
Overall	87155												0.846	0.862

AG: autonomic ganglia; CNS: central nervous system; PPI: protein-protein interaction; GOx: biological process interaction; NSCLC: non-small cell lung cancer; PWYx: pathway-pathway interaction; R^2: coefficient of determination; RECx: reaction-reaction interaction; RMSE: root-mean-square error; UAT: upper aerodigestive tract; $\#IC_{50}$: the number of drug responses; #Nodes: the number of nodes in the first and second hidden layers of neural networks; #Tours: the number of times to restart the fitting process.

Table S4 Prediction performances of using no interaction terms

Cancer type	$\#IC_{50}$	#All Features	#Drug Features	#Cancer features		#Nodes		#Tours	Performance	
				Residue	Others	1st	2nd		R^2	RMSE
AG	2971	62	39	0	23	62	8	200	0.861	0.74
Bone	3410	84	59	0	25	10	0	300	0.692	1.189
Breast	4706	129	77	9	43	26	6	200	0.901	0.649
CNS	4250	114	72	0	42	11	0	300	0.745	1.052
Cervix	1044	37	29	0	8	7	0	200	0.867	0.767
Endometrium	1073	33	25	0	8	11	4	200	0.698	1.18
Haematopoietic	4204	119	71	14	34	11	0	200	0.877	0.844
Kidney	2458	73	47	5	21	9	0	200	0.783	1.016
Large intestine	4628	141	73	38	30	12	0	300	0.732	1.106
Liver	1348	48	38	1	9	7	0	200	0.842	0.841
Lung (NSCLC)	9205	207	80	31	96	15	0	200	0.831	0.867
Lung (others)	7206	162	78	6	78	30	6	200	0.898	0.64
Lymphoid	13302	291	80	116	95	18	0	300	0.814	0.915
Oesophagus	3337	91	59	0	32	10	0	200	0.745	1.082
Ovary	3502	113	75	4	34	11	0	200	0.701	1.204
Pancreas	2421	84	63	0	21	10	0	200	0.856	0.815
Pleura	1431	36	25	0	11	11	4	200	0.824	0.852
Skin	5732	132	68	11	53	12	0	200	0.887	0.771
Soft tissue	1938	63	44	0	19	8	0	200	0.816	0.955
Stomach	2327	83	52	5	26	20	5	200	0.783	0.974
Thyroid	1352	33	27	0	6	6	0	300	0.696	1.293
UAT	3856	126	55	21	50	12	0	300	0.744	1.113
Urinary tract	1454	68	54	1	13	9	0	200	0.581	1.301
Overall	87155								0.817	0.940

AG: autonomic ganglia; CNS: central nervous system; NSCLC: non-small cell lung cancer; R^2: coefficient of determination; RMSE: root-mean-square error; UAT: upper aerodigestive tract; $\#IC_{50}$: the number of drug responses; #Nodes: the number of nodes in the first and second hidden layers of neural networks; #Tours: the number of times to restart the fitting process.

Table S5 Pathway enrichment analysis

PANTHER pathway	Reference list	Observed	Expected	Fold enrichment	P-value	FDR
Angiogenesis	173	6	0.38	15.83	2.46E-06	2.02E-04
Ras Pathway	74	4	0.16	24.67	2.53E-05	8.31E-04
Inflammation mediated by chemokine and cytokine signaling pathway	260	6	0.57	10.53	2.36E-05	9.69E-04
PDGF signaling pathway	148	5	0.32	15.42	2.05E-05	1.12E-03
Wnt signaling pathway	312	5	0.68	7.31	6.21E-04	1.70E-02
JAK/STAT signaling pathway	17	2	0.04	53.7	7.81E-04	1.83E-02
Cytoskeletal regulation by Rho GTPase	87	3	0.19	15.74	1.01E-03	2.06E-02
Axon guidance mediated by Slit/Robo	26	2	0.06	35.11	1.70E-03	3.11E-02
Interferon-gamma signaling pathway	29	2	0.06	31.48	2.09E-03	3.42E-02
Apoptosis signaling pathway	118	3	0.26	11.6	2.35E-03	3.51E-02
EGF receptor signaling pathway	134	3	0.29	10.22	3.34E-03	4.57E-02

FDR: false discovery rate.

Table S6 Drug-mutation interaction terms and their impact on IC_{50} in NSCLC cells

Interaction term	IC_{50} impact	$\|IC_{50}$ impact$\|$
PKA_102_CSV_X_Fingerprint_714	-1.8652	1.8652
PKA_260_HYD_X_Fingerprint_819	-1.5855	1.5855
PKA_247_HYD_X_Fingerprint_685	1.0754	1.0754
PKA_200_HYD_X_Fingerprint_673	0.7291	0.7291
PKA_197_B62_X_Fingerprint_576	0.5091	0.5091
**PKA_187_CHA_X_Fingerprint_791**	**-0.4563**	**0.4563**
PKA_112_POL_X_Fingerprint_659	-0.4440	0.4440
PKA_244_ENG_X_Fingerprint_576	0.3811	0.3811
PKA_73_ENG_X_Fingerprint_611	0.3802	0.3802
PKA_73_POL_X_Fingerprint_611	0.3772	0.3772
PKA_187_POL_X_Fingerprint_791	0.3621	0.3621
PKA_226_HYD_X_Fingerprint_576	0.3613	0.3613
PKA_293_X_Fingerprint_611	0.2765	0.2765
PKA_187_B62_X_Fingerprint_826	0.2702	0.2702
PKA_293_X_Fingerprint_647	-0.2575	0.2575
PKA_229_EXP_X_Fingerprint_576	0.2542	0.2542
PKA_197_EXP_X_Fingerprint_576	0.2540	0.2540
PKA_142_X_Fingerprint_611	-0.2385	0.2385
PKA_229_HYD_X_Fingerprint_576	0.2296	0.2296
PKA_270_POL_X_Fingerprint_576	0.2009	0.2009
PKA_270_HYD_X_Fingerprint_611	0.1979	0.1979
PKA_260_POL_X_Fingerprint_819	0.1119	0.1119
PKA_283_POL_X_Fingerprint_647	-0.1099	0.1099
PKA_280_ENG_X_Fingerprint_646	0.1027	0.1027
PKA_226_X_Fingerprint_644	0.0963	0.0963
PKA_293_EXP_X_Fingerprint_363	-0.0886	0.0886
PKA_73_ENG_X_Fingerprint_644	0.0852	0.0852
PKA_216_ASA_X_Fingerprint_646	-0.0618	0.0618
PKA_73_EXP_X_Fingerprint_702	-0.0443	0.0443
PKA_102_VOL_X_Fingerprint_714	-0.0433	0.0433
PKA_283_POL_X_Fingerprint_644	-0.0403	0.0403
PKA_160_HYD_X_Fingerprint_696	-0.0346	0.0346
PKA_175_ENG_X_Fingerprint_685	0.0342	0.0342
PKA_270_EXP_X_Fingerprint_611	0.0276	0.0276
PKA_252_ASA_X_Fingerprint_646	-0.0227	0.0227
PKA_283_ASA_X_Fingerprint_576	0.0202	0.0202
PKA_187_ASA_X_Fingerprint_791	-0.0192	0.0192
PKA_283_ASA_X_Fingerprint_647	0.0119	0.0119
PKA_197_VOL_X_Fingerprint_702	0.0118	0.0118
PKA_197_ASA_X_Fingerprint_798	0.0097	0.0097
**PKA_187_VOL_X_Fingerprint_826**	**-0.0090**	**0.0090**
PKA_123_VOL_X_Fingerprint_363	-0.0079	0.0079
PKA_77_ASA_X_Fingerprint_714	-0.0055	0.0055
PKA_73_CHA_X_Fingerprint_714	-0.0027	0.0027
PKA_283_VOL_X_Fingerprint_673	-0.0024	0.0024
PKA_283_ASA_X_Fingerprint_644	-0.0023	0.0023
PKA_270_VOL_X_Fingerprint_673	-0.0004	0.0004

The features illustrated in Figure 4 are highlighted in bold.

Table S7 Comparison between full training sets' features and reduced sets' features

	All selected features				Selected interaction terms			
	Full set	Reduced set	Overlap		Full set	Reduced set	Overlap	
			Count	%*			Count	%*
AG	62	44	43	69.4	22	14	14	63.6
Bone	84	81	74	88.1	19	20	17	89.5
Breast	129	136	108	83.7	28	30	20	71.4
CNS	114	102	90	78.9	26	22	18	69.2
Cervix	37	32	31	83.8	5	3	3	60.0
Endometrium	33	26	22	66.7	8	6	5	62.5
Haematopoietic	119	137	98	82.4	34	41	20	58.8
Kidney	73	64	58	79.5	19	18	15	78.9
Large intestine	141	152	120	85.1	64	61	54	84.4
Liver	48	32	31	64.6	9	6	6	66.7
Lung (NSCLC)	207	201	174	84.1	93	98	78	83.9
Lung (others)	162	166	130	80.2	86	93	63	73.3
Lymphoid	291	262	223	76.6	135	124	107	79.3
Oesophagus	91	96	78	85.7	16	22	11	68.8
Ovary	113	118	102	90.3	29	29	24	82.8
Pancreas	84	87	77	91.7	17	19	13	76.5
Pleura	36	43	32	88.9	8	7	6	75.0
Skin	132	99	91	68.9	38	28	23	60.5
Soft tissue	63	59	55	87.3	8	8	8	100.0
Stomach	83	73	62	74.7	21	16	13	61.9
Thyroid	33	32	31	93.9	3	3	3	100.0
UAT	126	127	106	84.1	61	58	44	72.1
Urinary tract	68	68	60	88.2	16	15	12	75.0
Overall	2329	2237	1896	81.4	765	741	577	75.4

%*: defined by the overlap count dividing the full set's feature count.

Table S8 Prediction performances of the QSMART model with neural networks in reduced sets

Cancer type	#IC_{50}	#All	#Drug	#Cancer features		#Interactions		#Nodes		#Tours	Performance	
		Features	Features	Residue	Others	DxM	Others	1st	2nd		R^2	RMSE
AG	2674	44	27	0	3	1	13	44	7	200	0.823	0.824
Bone	3069	81	48	0	13	4	16	9	0	300	0.824	0.906
Breast	4236	136	75	0	31	14	16	27	6	200	0.9	0.648
CNS	3825	102	56	0	24	9	13	11	0	300	0.885	0.703
Cervix	940	32	25	0	4	1	2	6	0	200	0.82	0.891
Endometrium	966	26	17	0	3	4	2	9	3	200	0.766	1.023
Haematopoietic	3784	137	64	5	27	33	8	12	0	200	0.83	0.994
Kidney	2213	64	41	0	5	0	18	8	0	200	0.731	1.132
Large intestine	4166	152	64	15	12	47	14	13	0	300	0.826	0.891
Liver	1214	32	23	0	3	1	5	7	0	200	0.792	0.953
Lung (NSCLC)	8285	201	68	2	33	44	54	15	0	200	0.846	0.828
Lung (others)	6486	166	56	3	14	48	45	31	6	200	0.871	0.72
Lymphoid	11972	262	71	46	21	72	52	17	0	300	0.863	0.783
Oesophagus	3004	96	58	0	16	6	16	10	0	200	0.885	0.733
Ovary	3152	118	70	3	16	9	20	11	0	200	0.729	1.143
Pancreas	2179	87	60	0	8	0	19	10	0	200	0.675	1.236
Pleura	1288	43	32	0	4	0	7	13	4	200	0.877	0.698
Skin	5159	99	49	2	20	8	20	10	0	200	0.806	1.018
Soft tissue	1745	59	41	0	10	2	6	8	0	200	0.838	0.894
Stomach	2095	73	42	3	12	11	5	18	5	200	0.839	0.824
Thyroid	1217	32	24	0	5	0	3	6	0	300	0.831	0.96
UAT	3471	127	52	1	16	6	52	12	0	300	0.767	1.067
Urinary tract	1309	68	48	0	5	7	8	9	0	200	0.851	0.766
Overall	78449										0.839	0.881

AG: autonomic ganglia; CNS: central nervous system; DxM: drug-mutation interaction term; NSCLC: non-small cell lung cancer; R^2: coefficient of determination; RMSE: root-mean-square error; UAT: upper aerodigestive tract; #IC_{50}: the number of drug responses; #Nodes: the number of nodes in the first and second hidden layers of neural networks; #Tours: the number of times to restart the fitting process.

Table S9 Cancer cell line features

Feature level	Feature	Nomenclature	Value
Residue	PKA position	PKA_[POSITION]	$x_i = \sum_{k=1}^{K} M_{ki}\omega, \omega = \{1, CSV_{ki}, EXP_k\}$
	Mutant type	PKA_[POSITION]_[MT]	$x_{im} = \sum_{k=1}^{K} M_{kim}\omega, \omega = \{1, CSV_{ki}, EXP_k\}$
	Charge	PKA_[POSITION]_CHA	$x_i = \sum_{k=1}^{K} C_{ki}$
	Polarity	PKA_[POSITION]_POL	$x_i = \sum_{k=1}^{K} P_{ki}$
	Hydrophobicity	PKA_[POSITION]_HYD	$x_i = \sum_{k=1}^{K} H_{ki}$
	Accessible surface area	PKA_[POSITION]_ASA	$x_i = \sum_{k=1}^{K} A_{ki}$
	Side-chain volume	PKA_[POSITION]_VOL	$x_i = \sum_{k=1}^{K} V_{ki}$
	Energy per residue	PKA_[POSITION]_ENG	$x_i = \sum_{k=1}^{K} E_{ki}$
	Substitution score	PKA_[POSITION]_B62	$x_i = \sum_{k=1}^{K} S_{ki}$
Motif	Sequence motif	MOT_2D_[NAME]	$x_t = \sum_{k=1}^{K} \sum_{n=1}^{N_k} M_{kn} L_t(k,n)\omega, \omega = \{1, CSV_{kn}, EXP_k\}$
	Structural motif	MOT_3D_[NAME]	$x_T = \sum_{k=1}^{K} \sum_{n=1}^{N_k} M_{kn} L_T(k,n)\omega, \omega = \{1, CSV_{kn}, EXP_k\}$
Domain	Subdomain	SDOM_[NAME]	$x_d = \sum_{k=1}^{K} \sum_{n=1}^{N_k} M_{kn} L_d(k,n)\omega, \omega = \{1, CSV_{kn}, EXP_k\}$
	Functional domain	DOM_[NAME]	$x_D = \sum_{k=1}^{K} \sum_{n=1}^{N_k} M_{kn} L_D(k,n)\omega, \omega = \{1, CSV_{kn}, EXP_k\}$
Gene	Mutation	MUT_[GENE]	$x_k = M_k\omega = \sum_{n=1}^{N_k} M_{kn}\omega, \omega = \{1, CSV_{kn}, EXP_k\}$
	Expression	EXP_[GENE]	$x_k = EXP_k$, from GDSC
	Copy number variation	CNV_[GENE]	$x_k = CNV_k = \{gain, neutral, loss\}$, from COSMIC
Family	Family	SFAM_[NAME]	$x_f = \sum_{k=1}^{K} M_k F_f(k)\omega = \sum_{k=1}^{K} \sum_{n=1}^{N_k} M_{kn} F_f(k)\omega, \omega = \{1, CSV_{kn}, EXP_k\}$
	Group	FAM_[NAME]	$x_g = \sum_{k=1}^{K} M_k G_g(k)\omega = \sum_{k=1}^{K} \sum_{n=1}^{N_k} M_{kn} G_g(k)\omega, \omega = \{1, CSV_{kn}, EXP_k\}$
Pathway	Reaction	REC_[REACTOME_ID]	$x_r = \sum_{k=1}^{K} M_k R_r(k)\omega = \sum_{k=1}^{K} \sum_{n=1}^{N_k} M_{kn} R_r(k)\omega, \omega = \{1, CSV_{kn}, EXP_k\}$
	Pathway	PWY_[REACTOME_ID]	$x_w = \sum_{k=1}^{K} M_k W_w(k)\omega = \sum_{k=1}^{K} \sum_{n=1}^{N_k} M_{kn} W_w(k)\omega, \omega = \{1, CSV_{kn}, EXP_k\}$
	Biological process	GO_[GO_ID]	$x_b = \sum_{k=1}^{K} M_k B_b(k)\omega = \sum_{k=1}^{K} \sum_{n=1}^{N_k} M_{kn} B_b(k)\omega, \omega = \{1, CSV_{kn}, EXP_k\}$
Sample	Primary site	CLS_Primary_site	From COSMIC
	Site subtype 1	CLS_Site_subtype_1	
	Site subtype 2	CLS_Site_subtype_2	
	Site subtype 3	CLS_Site_subtype_3	
	Primary histology	CLS_Primary_histology	
	Histology subtype 1	CLS_Histology_subtype_1	
	Histology subtype 2	CLS_Histology_subtype_2	
	Histology subtype 3	CLS_Histology_subtype_3	
	Microsatellite instability	CLS_msi	
	Average ploidy	CLS_average_ploidy	
	Tumour source	CLS_tumour_source	
	Age	CLS_age	
	Gender	CLS_gender	
	NCI code	CLS_NCI_code	

M_{ki}: if the residue of protein kinase k aligned to PKA position i is mutated (1) or not (0); CSV_{ki}: the conservation score of the residue of protein kinase k aligned to PKA position i; EXP_k: the gene expression level of protein kinase k; M_{kim}: if the residue of protein kinase k aligned to PKA position i is mutated to the amino acid type m (1) or not (0); C_{ki}, P_{ki}, H_{ki}, A_{ki}, V_{ki}, or E_{ki}: respectively mean the charge, polarity, hydrophobicity, accessible surface area, side-chain volume, or energy differences caused by the mutated residue of protein kinase k aligned to PKA position i; S_{ki}: the BLOSUM62 substitution score of the mutated residue of protein kinase k aligned to PKA position i; N_k: the length of protein kinase k sequence; M_{kn}: if the nth residue of protein kinase k is mutated (1) or not (0); $L_t(k,n)$, $L_T(k,n)$, $L_d(k,n)$, or $L_D(k,n)$: respectively mean if the nth residue of protein kinase k is located in sequence motif t, structural motif T, subdomain d, or functional domain D (1) or not (0); CSV_{kn}: the conservation score of the nth residue of protein kinase k; CNV_k: the copy number variation status of protein kinase k; $F_f(k)$ or $G_g(k)$: respectively mean if protein kinase k belongs to family f or group p (1) or not (0); $R_r(k)$, $W_w(k)$, or $B_b(k)$: respectively mean if protein kinase k is implicated in reaction r, pathway w, or biological process b (1) or not (0); NCI code: National Cancer Institute (NCI) Thesaurus code.

Table S10 Remaining drug features of feature screening with a reversed feature prioritization

Drug feature	VIF
ALogP	4.72554
ALogp2	4.11270
AMR	4.99997
BCUTw-1l	1.37218
BCUTw-1h	1.54648
BCUTc-1l	3.02942
BCUTc-1h	2.78522
BCUTp-1l	2.40230
BCUTp-1h	2.11239
PNSA-1	2.64945
PNSA-3	4.54709
RPCS	1.84704
RNCS	1.91853
Wlambda2.unity	2.65717
Weta2.unity	1.30732
Weta3.unity	1.78801
nAcid	1.19615
ATSc3	2.61343
ATSc4	2.38344
nBase	1.44670
C1SP1	1.28125
C2SP1	1.16451
C4SP3	1.73272
SCH-3	4.64373
SCH-4	4.99948
SCH-5	1.59229
nHBDon	4.45565
khs.dCH2	1.30666
khs.ssS	1.50674
Fingerprint_346	2.50190
Fingerprint_476	1.63186
Fingerprint_500	1.48860
Fingerprint_820	1.56166

VIF: variance inflation factor.

Table S11 Prediction performances of using split QSMART models with neural networks

Cancer type	#Nodes		Split QSMART models						Performance comparison		
			Drug		Cancer cell line		Interaction		Full model	Split models	Difference
	1st	2nd	#Features	R^2_{Drug}	#Features	R^2_{Cancer}	#Features	$R^2_{Interaction}$	R^2_{Full}	R^2_{SSP}	R^2_{Full}-R^2_{SSP}
AG	62	8	31	0.641	9	0.042	22	0.009	0.879	0.692	0.187
Stomach	20	5	49	0.611	13	0.053	21	0.062	0.836	0.726	0.110
Breast	26	6	70	0.629	31	0.070	28	0.073	0.880	0.771	0.109
Pleura	11	4	23	0.614	5	0.043	8	0.061	0.805	0.718	0.088
Haematopoietic	11	0	58	0.599	27	0.092	34	0.098	0.858	0.789	0.070
Oesophagus	10	0	58	0.699	17	0.027	16	0.050	0.841	0.776	0.066
Soft tissue	8	0	45	0.561	10	0.100	8	0.104	0.818	0.765	0.053
Cervix	7	0	65	0.683	23	0.072	26	0.055	0.858	0.810	0.048
Liver	7	0	35	0.652	4	0.020	9	0.126	0.836	0.798	0.038
Urinary tract	9	0	47	0.673	5	0.105	16	0.048	0.863	0.826	0.037
Lung (NSCLC)	15	0	72	0.610	42	0.084	93	0.128	0.854	0.822	0.031
Skin	12	0	64	0.685	30	0.041	38	0.122	0.875	0.848	0.027
Bone	10	0	52	0.607	13	0.111	19	0.112	0.856	0.830	0.026
Lung (others)	30	6	58	0.610	18	0.121	86	0.104	0.859	0.834	0.024
UAT	12	0	50	0.727	15	0.062	61	0.085	0.881	0.873	0.008
Pancreas	10	0	60	0.717	7	0.058	17	0.061	0.833	0.835	-0.002
Thyroid	6	0	25	0.713	5	0.067	3	0.053	0.830	0.833	-0.003
Endometrium	11	4	21	0.709	4	0.076	8	0.099	0.878	0.884	-0.006
Ovary	11	0	64	0.648	20	0.092	29	0.122	0.844	0.861	-0.017
Kidney	9	0	51	0.666	3	0.074	19	0.126	0.836	0.866	-0.030
Lymphoid	18	0	72	0.661	84	0.097	135	0.149	0.873	0.907	-0.034
CNS	11	0	29	0.669	3	0.033	5	0.244	0.864	0.946	-0.081
Large intestine	12	0	53	0.574	24	0.160	64	0.209	0.814	0.943	-0.129
Overall				0.663		0.126		0.152	0.863	0.940	-0.077

R^2_{Full}: the performance of using a full QSMART model with neural networks shown in Table S2; R^2_{SSP}: the sum of split model performances ($R^2_{SSP} = R^2_{Drug} + R^2_{Cancer} + R^2_{Interaction}$). AG: autonomic ganglia; CNS: central nervous system; NSCLC: non-small cell lung cancer; UAT: upper aerodigestive tract; #Nodes: the number of nodes in the first and second hidden layers of neural networks.
